# Tubulointerstitial nephritis antigen-like 1 promotes the progression of liver fibrosis after HCV eradication with direct-acting antivirals

**DOI:** 10.7150/ijbs.103305

**Published:** 2025-01-01

**Authors:** Lei Lei, Hu Li, Xue-Kai Wang, Jian-Rui Li, Han Sun, Hong-Ying Li, Jia-Yu Li, Mei Tang, Jing-Chen Xu, Biao Dong, Yue Gong, Dan-Qing Song, Jian-Dong Jiang, Zong-Gen Peng

**Affiliations:** 1 CAMS Key Laboratory of Antiviral Drug Research, Institute of Medicinal Biotechnology, Chinese Academy of Medical Sciences & Peking Union Medical College, Beijing, 100050, China.; 2 NHC Key Laboratory of Biotechnology for Microbial Drugs, Institute of Medicinal Biotechnology, Chinese Academy of Medical Sciences & Peking Union Medical College, Beijing 100050, China.; 3 Beijing Key Laboratory of Antimicrobial Agents, Institute of Medicinal Biotechnology, Chinese Academy of Medical Sciences & Peking Union Medical College, Beijing, 100050, China.; 4 State Key Laboratory of Bioactive Substances and Functions of Natural Medicines, Institute of Medicinal Biotechnology, Peking Union Medical College and Chinese Academy of Medical Sciences, Beijing 100050, China.

**Keywords:** TINAGL1, HCV eradication, DAAs, Liver fibrosis, PDGF-BB/PDGFRβ pathway

## Abstract

Although therapies based on direct-acting antivirals (DAAs) effectively eradicate hepatitis C virus (HCV) in patients, there is still a high risk of liver fibrosis even after a sustained virological response. Therefore, it is of great clinical importance to understand the mechanism of potential factors that promote liver fibrosis after virological cure by treatment with DAAs. Here, we found that tubulointerstitial nephritis antigen-like 1 (TINAGL1) is significantly increased in HCV-infected hepatocytes and in the liver of patients with liver fibrosis, and that higher TINAGL1 expression persists in HCV-eradicated hepatocytes after treatment with DAAs. Overexpression of TINAGL1 in the liver triggers and exacerbates liver fibrosis, and xenotransplantation of HCV-eradicated Huh7.5 cells leads to a higher risk of hepatocellular carcinoma. Conversely, knockdown of TINAGL1 expression prevents and attenuates the progression of liver fibrosis in mice. TINAGL1 binds and stabilizes platelet-derived growth factor-BB (PDGF-BB) in hepatocytes, leading to an increase in intracellular and extracellular PDGF-BB, which sensitizes the PDGF-BB/PDGFRβ pathway to activate hepatic stellate cells. This study highlights that TINAGL1 is a new factor contributing to liver fibrosis after injury, including but not limited to HCV infection, even after virological cure by DAAs, and emphasizes the therapeutic potential of TINAGL1 as an innovative target for the treatment of liver fibrosis.

## Introduction

Infection with hepatitis C virus (HCV), a positive single-stranded RNA virus, causes acute and chronic hepatitis that easily progresses to liver fibrosis, cirrhosis, and even hepatocellular carcinoma (HCC) 1. According to the WHO reports, 58 million individuals were living with chronic HCV infection in 2019, resulting in 399,000 deaths per year worldwide 2-4. However, only 20% of HCV-infected patients were diagnosed and 13% of HCV-infected patients were treated 5. In the clinic, most HCV-infected patients are treated with direct-acting antivirals (DAAs), which can achieve efficient HCV eradication 6. However, those patients remain at high risk of progressing to fibrosis or cirrhosis that leads to HCC, even after a sustained virological response (SVR) 7-14.

Liver fibrosis is a common pathologic process in liver diseases, caused by various etiologies such as viral hepatitis, alcoholic steatohepatitis, and non-alcoholic steatohepatitis 15, 16. Extracellular matrix (ECM) such as collagens and fibronectin secreted by activated myofibroblasts in the liver play a critical role in the progression of liver fibrosis 17, 18. Among them, matricellular proteins are essential for the development of liver fibrosis 19. When the liver is damaged, matricellular proteins accumulate excessively, which further activates fibroblasts and triggers fibrosis, leading to cirrhosis and even HCC 19. Several studies have reported that fibrosis-related signals such as extracellular vesicle modification (downregulation of antifibrogenic miRNAs and upregulation of fibrogenic proteins) 7 and HCC risk (epigenetic changes) 9 persist in HCV-infected patients after SVR with DAAs. However, the detailed mechanism remains to be further clarified, and there is still no structural and experimental evidence that these factors contribute to the occurrence of liver fibrosis and have therapeutic potential, to date, no specific drug has been approved for the treatment of liver fibrosis 20. Therefore, elucidating the molecular mechanism of the factors that drive the progression of liver fibrosis after HCV elimination by treatment with DAAs will contribute to identifying new therapeutic targets.

In the search for such factors, we found that tubulointerstitial nephritis antigen-like 1 (TINAGL1) could be a new profibrotic factor. TINAGL1 is an ECM protein 21, also known as lipocalin7 22, adrenocortical zonation factor-1 21, and tubulointerstitial nephritis antigen-related protein 23, which was first identified in the kidney in 2001 24. Later studies reported in 2007 that TINAGL1 regulates cell adhesion by interacting with integrin receptors on the cell membrane 21. In addition, TINAGL1 is thought to play a role in the pathology of many cancers, including HCC through the transforming growth factor-beta1 (TGF-β1)/small mothers against decapentaplegic protein (SMAD)/vascular endothelial-derived growth factor axis 25, non-small cell lung cancer 26, gastric cancer by regulating multiple matrix metallopeptidase expression 27, and breast cancer through integrin/focal adhesion kinase and epidermal growth factor receptor signaling 28, 29. Recent research has shown that TINAGL1 plays a role in gastric lesions caused by bacterial infections and positively correlates with disease progression 30. Importantly, TINAGL1 can directly bind to SMAD4 to exacerbate intestinal fibrosis 31. However, the role of TINAGL1 in liver fibrosis is still unknown.

In this study, we demonstrated for the first time the correlation and mechanism between TINAGL1 and liver fibrosis in patients and mice and at the cellular level. Significantly, TINAGL1 is proposed as a profibrotic factor in HCV-infected patients after virological cure by treatment with DAAs and showed promising therapeutic potential in controlling liver fibrosis, providing an innovative target for the treatment of liver fibrosis after liver injury, including but not limited to HCV infection.

## Materials and Methods

### Animal models

Animal experiments were conducted in accordance with the National Guidelines for Housing and Care of Laboratory Animals. The Institutional Animal Care and Use Committee of the Institute of Medicinal Biotechnology & Chinese Academy of Medical Sciences (SYXK (Jing) 2017-0023) approved this research.

Six- to eight-week-old male C57BL/6J mice (20 - 22 g) and BALB/c nude mice (20 - 22 g) with SPF grade were obtained from Beijing SPF Biotechnology Co., Ltd (Beijing, China). Male Sprague-Dawley (SD) rats (150 - 170 g) with SPF grade were obtained from Beijing HuaFuKang Biological Technology Co., Ltd (Beijing, China). All animals were maintained under SPF conditions, including a 12 - hour light - dark cycle, and free access to water and food.

For the carbon tetrachloride (CCl_4_)-induced liver fibrosis model, C57BL/6J mice were intraperitoneally injected with olive oil (8001-23-0, Shanghai Macklin Biochemical Co., Ltd, China) as vehicle control, or with 0.75 mL/kg of CCl_4_ (Tianjin Fuchen Chemical Reagent Factory, China, dissolved in olive oil, in a ratio of 1 : 9) twice a week for six to eight weeks. After the treatment period, liver tissues and serum samples were harvested and preserved at -80°C for further analysis.

For the diethylnitrosamine (DEN)-induced liver fibrosis model, male SD rats were treated with 100 ppm DEN (N0756, Sigma-Aldrich) in drinking water for seven weeks to induce moderate fibrosis, and then treated with 30 ppm DEN for another five weeks. Liver tissues were collected and stored at -80°C for subsequent analysis 32.

### Tumor xenograft experiment

2 × 10^6^ cells of HCV-eradicated Huh7.5 cells, DAAs-treated Huh7.5 cells, or native control Huh7.5 cells were subcutaneously xeno-implanted into the flanks of BALB/c nude mice. Tumor growth was monitored every three days by measuring each tumor size with a vernier caliper, and the volume was calculated by: V= (length×width^2^)/2. The tumor mass was weighed at the end of the experiment.

### Construction and infection of adenovirus vector

Adeno-associated virus 8 (AAV8) containing *Tinagl1* was prepared by Hanbio Biotechnology CO., Ltd (Shanghai, China). Specifically, the pHBAAV-CMV-MCS-EF1-ZsGreen vector was used for cloning the full coding sequences of mouse *Tinagl1*-*His*. For downregulating TINAGL1 expression in mouse livers, the pHBAAV-U6-MCS-CMV-EGFP vector was used to clone mouse-specific short hairpin RNAs (shRNAs) targeting the *Tinagl1* gene. The specific shRNA sequences for *Tinagl1* were 5'-CCTGTTCAAGCACTCATGGAA-3', and the control shRNA sequences were 5'-TTCTCCGAACGTGTCACGTAA-3'. Recombinant adenoviruses had a titer of 1 × 10^12^ viral genomes per milliliter. For the *in vivo* experiments, six- to eight-week-old male C57BL/6J mice were intravenously injected with 100 μL of the viruses and then intraperitoneally administered with or without CCl_4_ (0.75 mL/kg body weight, twice a week) for six or eight weeks.

### Immunohistochemistry staining

Immunohistochemistry (IHC) was conducted on sections of paraffin-embedded tissues. Sections were sourced from human liver tissue microarray slides (D107Lv01, Bioaitech, Xian, China). Details on patient information are provided in **Table S1**. TINAGL1 expression was detected using a rabbit polyclonal antibody against TINAGL1 (1:150 dilution, Proteintech Cat# 12077-1-AP, RRID: AB_2058942). The tissue sections were incubated with primary antibodies overnight at 4°C and subsequently with an HRP-conjugated secondary antibody. Visualization of IHC staining on the liver tissue microarray slides was achieved using CaseViewer 2.4 software. Staining intensity was scored on a scale ranging from 0 (negative), 1 (weak), 2 (moderate), to 3 (strong). The H-Score was used to quantify staining intensity (H-SCORE = Σ(PI×I) = percentage of cells of weak intensity×1 + percentage of cells of moderate intensity×2 + percentage of cells of strong intensity×3).

Hematoxylin and eosin, Masson, and Sirius Red staining were performed on mouse liver samples to visualize liver morphology to assess liver fibrosis stages, which was semi-quantitated by ImageJ software.

### Human cytokine microarray

Cytokine concentrations in the cell medium of Huh7.5 cells transfected with the TINAGL1 plasmid were quantified using the human Cytokine Array G5 (Cat# AAH-CYT-G5-8, RayBiotech), which contains 80 different human cytokine antibodies. Huh7.5 cells were seeded into 6-well plates and then transfected with the TINAGL1 plasmid, and the medium was replaced with serum-free medium after 24 hours. The supernatant medium was collected and filtered when the cells were 90% - 100% confluent. After incubation with the supernatant medium, the arrays were processed and sent to Raybiotech for scanning and quantification of the signals. Cytokine signals were scanned using the InnoScan 300 Microarray Scanner (Innopsys, Carbonne, France) with Cy3 channel at a wavelength of 532 nm. Q-Analyzer software was used to process the raw fluorescence data, and the background fluorescence intensities were used to normalize the fluorescence data of each spot. Cytokines with a ratio of at least 1.20 were considered upregulated, and those with a ratio of 0.83 or less were considered downregulated. Expression fold-change ratios between 1 - 1.19 and 0.84 - 0.99 are classified as no difference. All human cytokine antibodies are available in **Table S2**.

### Cell culture

Dr. Hong-Wei He (Peking Union Medical College, Beijing, China) generously provided LX-2 cells, which are human immortalized hepatic stellate cells. LX-2 cells were maintained in Dulbecco's modified Eagle's medium Gluta MAX-I (10566-016, Gibco, USA) with 10% FBS (AQ-mv-09900, Beijing Aoqing Biotech Co., Ltd., China) and 1% penicillin-streptomycin (PS, 15140-122, Gibco, USA). Huh7.5 cells, HEK293T cells, and Hep G2 cells were cultured in Dulbecco's modified Eagle's medium (C11995500BT, Gibco, USA) supplemented with 10% FBS (16000-044, Gibco, USA) and 1% PS. Hep G2.2.15 cells were cultured in Dulbecco's modified Eagle's medium supplemented with 10% FBS, 1% PS, and 400 μg/mL Geneticin.

### HCV virus infection

Huh7.5 cells were infected with HCV virus genotype 2a JFH-1/J6 for a specified duration. Intracellular RNAs and proteins were extracted with RNeasy Mini Kit (169041472, QIAGEN) and detected by qRT-PCR. HCV viral stock was prepared as previously described 33.

### Construction of HCV-eradicated Huh7.5 cells

Huh7.5 cells were infected with a HCV viral stock (MOI = 0.1) for nine days and then treated with a fixed dose of DAAs drugs (1 μM simeprevir, 1 nM daclatasvir, and 5 μM sofosbuvir). Subsequently, these cells were passaged every three days. Following the completion of a nine-day DAAs treatment, the cells were passaged and cultured in a normal cell culture medium without DAAs up to thirty-six days, with monitoring of the intracellular HCV RNA levels every three days 34.

### Co-immunoprecipitation (CO-IP)

HEK293T cells and Huh7.5 cells transfected with the *TINAGL1-Flag* and *PDGF-BB-HA* were collected in a 1.5 mL EP tube when they reached 100 % confluence in a 10-cm dish. 1 mL of IP lysis buffer (87788, ThermoFisher) with complete protease inhibitor cocktail was added, placed on ice for 30 minutes, and centrifuged at 12,000 ×g for 20 minutes. 100 μL of the supernatant was transferred to a new tube as input, and the rest was incubated overnight at 4°C in an orbital roller with the pre-washed HA magnetic beads (88838, Thermo Scientific, USA) or Flag magnetic beads (A36797, ThermoFisher). After washing five times with ice-cold IP lysis buffer, the beads were resuspended by briefly vortexing with 100 μL 1× reducing sample buffer (39000, ThermoFisher) and then incubated in a heat block at 100°C for 10 minutes. The proteins are detected by Western blot using primary rabbit monoclonal antibody against HA, mouse monoclonal antibody against Flag, or rabbit polyclonal antibody against GAPDH.

### Surface plasmon resonance (SPR) assay

SPR measurements were carried out using a Reichert 4SPR System with SR7000 Gold Sensor Slide Ni-NTA Surface chip (13206063, Reichert, USA). 60 μg of recombinant human TINAGL1 protein (rhTINAGL1, C - 6× His, Cat# CB22, Novoprotein, China) was immobilized in parallel flow channels of the Ni-NTA Surface chip with 40 mM Nickel Sulfate (656895, Sigma, USA). Recombinant human PDGF-BB (rhPDGF-BB) protein (Cat# 100-14B, Peprotech, USA) was dissolved in PBST, and a dilution series of rhPDGF-BB was injected into the flow system at a flow rate of 62.5 μL/minutes. The association time was 4 minutes, and the dissociation time was 5 minutes.

For drug screening, the compound database contains 1760 FDA-approved drugs and 640 natural products obtained from Topscience (L6000, https://www.tsbiochem.com/library). Recombinant human TINAGL1 protein was immobilized and compounds were diluted to 10 μM with PBST in a 96-well measuring plate, and then passed through the Ni-NTA chip coupled to the TINAGL1 protein at a flow rate of 62.5 μL/min. The association time was 4 minutes, and the dissociation time was 5 minutes. For these drugs capable of binding to TINAGL1, each compound was diluted 5 times from 50 μM to 1.5625 μM, and then passed through the Ni-NTA chip from low concentration to high concentration. All solutions were sterilized by filtering through 0.22 μm poresized filters and degassed at room temperature. Binding kinetics were analyzed using TraceDrawer v1.8.1 software.

### Co-localization assay of TINAGL1 and PDGF-BB

HEK293T cells were plated in 6-well plates with coverslips and transfected with the *TINAGL1-Flag* and *PDGF-BB-HA* plasmid for 72 hours. Cells were fixed with 4% paraformaldehyde for 20 min and permeabilized with 0.3% Triton X-100 for 20 min. After washing, cells were incubated with Flag (1 : 800, Proteintech Cat# 66008-4-Ig, RRID:AB_2918475) and HA (1 : 1000, Cell Signaling Technology Cat# 3724 (also 3724S), RRID:AB_1549585) antibodies overnight at 4°C. Then cells were rinsed with PBS and incubated for 1 h with secondary antibodies conjugated with AF488 (1:200, TRANS, R21205). Cells were observed using a Leica TCS SP8 confocal microscope with stimulated emission depletion (STED) at 3× super resolution (Leica Microsystems GmbH, Mannheim, Germany) for green fluorescence of PDGF-BB, and red fluorescence of TINAGL1. Statistical analysis of co-localization of TINAGL1 and PDGF-BB fluorescence intensities was performed using ImageJ software.

### Protein half-life assay

Huh7.5 cells were plated in 6-well plates and transfected with the *TINAGL1-Flag* plasmid for 48 hours. The cells were then treated with 100 mg/mL of cycloheximide (HY-12320, MedChem Express, USA) for 0, 2, 4, 6, 8, and 10 hours. Cell lysates were collected for measurement of PDGF-BB protein levels by Western blot.

### Protein modeling analysis

Protein modeling analysis was built using the SWISS-MODEL (https://swissmodel.expasy.org/) with the TINAGL1 protein (UniProt ID: Q9GZM7, using the included AlphaFold 2 prediction of the three-dimensional protein structure) and PDGF-BB protein (UniProt ID: P01127, using the included AlphaFold 2 prediction of the three-dimensional protein structure). A protein docking study between three-dimensional protein structures of protein PDGF-BB and TINAGL1 was carried out using Discovery Studio 4.5 software. ZDock software was employed. RDock software was used to further optimize the results of protein docking. The highest ranked docking conformation was considered the binding conformation.

### mRNA microarray

Huh7.5 cells at different time points of HCV infection (one, two, and three months) were selected to perform mRNA microarray by Kangcheng Biotech Co., Ltd. (Shanghai, China) using Agilent chips (Agilent Technologies, Santa Clara, CA, USA). RNA samples were conducted as previously described (RNA Preparation method). Significantly differentially expressed genes (fold change ≥ 2) were used for further analysis.

### Liver hydroxyproline (HYP) assay

Liver samples were obtained immediately after the mice were sacrificed and were subjected to a modified acid hydrolysis protocol to determine the hydroxyproline concentration using a hydroxyproline assay kit (A030-3, Nanjing Jiancheng Bioengineering Institute, China).

### Serum biochemistry

Serum levels of alanine aminotransferase (ALT) and aspartate aminotransferase (AST) were measured using ALT and AST assay kit according to the manufacturer's instruction (C009-2-1 and C010-2-1, Nanjing Jiancheng Bioengineering Institute, China).

### rhTINAGL1 treatment

LX-2 cells were seeded in 12-well plates and stimulated for 48 hours with fresh serum-free medium with or without rhTINAGL1 (Cat# CB22, Novoprotein) in a concentration gradient. The cells were collected for real-time quantitative PCR.

### RNA preparation and real-time quantitative PCR (qRT-PCR)

Total RNA of the cell culture sample and liver tissues were extracted using the Total RNA kit (R4011-03, Magen, China). The RNA concentration was determined using a NanoDrop 2000 spectrophotometer (Thermo Scientific, USA). RNAs were detected using the HiScript II One Step qRT-PCR SYBR Green Kit (Q221-01, Vazyme).

For quantification of HCV RNA, total RNA was extracted from Huh7.5 cells using a RNeasy Mini Kit (169041472, QIAGEN) and detected using the AgPath-ID One-Step RT-PCR Kit (4388520, Applied Biosystems). mRNA levels of specific genes were quantified to GAPDH using the 2^-ΔΔCt^ method. All analyses were performed using an ABI 7500 Fast Real-Time PCR system (Applied Biosystems, Naerum, Denmark). All primers for qRT-PCR are available in **Table S3**.

### Gene silencing

The sequences of siRNA that specifically target TINAGL1 or PDGFRB (RIBOBIO, China) are shown in **Table S4**. Huh7.5 cells were transfected with either control siRNA or 50 nM TINAGL1 siRNA, or LX-2 cells were transfected with control siRNA or 50 nM PDGFRB siRNA using the lipofectamine RNAiMAX reagent (56532, Invitrogen). Cells were collected at 48 or 72 hours for RNA and protein extract and analysis.

### Preparation of the conditioned medium

Huh7.5 cells were seeded in 6-well plates and then transfected with the TINAGL1 plasmids for 8 hours. After incubation for 24 hours, the cells were washed three times with serum-free medium and cultured for 48 hours. The culture supernatants were collected and then centrifugated at 10,000 ×g for 10 minutes at 4°C. The supernatants were added to culture LX-2 cells or frozen at -80°C until use. Cells were lysed and processed for either RNA isolation or protein lysate preparation at 48 hours.

### Co-culture of Huh7.5 cells and LX-2 cells

A transwell co-culture system was utilized in this study. Huh7.5 cells infected with or without HCV, or transfected with or without the TINAGL1 plasmid were seeded in 12-well plates for 24 hours. LX-2 cells transfected with or without siRNA for PDGFRB were seeded in transwell inserts (3401, Corning) and then placed into the wells with Huh7.5 cells. After 48 hours of co-culture, both supernatants and cells were harvested for analysis.

### Western blot

Proteins were extracted from mouse liver tissues using T-PER lysis buffer (78510, ThermoFisher) supplemented with a protease inhibitor cocktail (78443, ThermoFisher) or from cells using M-PER lysis buffer (78505, ThermoFisher) also with a protease inhibitor cocktail. Protein concentrations were determined with a BCA protein assay kit (1863381, ThermoFisher). An equal amount of protein was denatured at 100°C for 10 minutes, separated with SDS-PAGE, and transferred to polyvinylidene fluoride (PVDF) membranes. The membranes were blocked with 5% non-fat dry milk in TBST for 1 hour and then incubated with the primary antibodies overnight at 4°C. After being washed with TBST, the membranes were incubated with secondary horseradish peroxidase (HRP)-conjugated antibodies for 1 hour at room temperature. Protein expression signals were detected using a ChemiDoc MP Imaging System (Bio-Rad, USA). ACTB (1:5000, Huabio Cat# ET1702-52, RRID: AB_3070314) and GAPDH (1 : 5000, Proteintech Cat# 60004-1-Ig, RRID: AB_2107436) were used for normalization, and densitometric analysis of each band was performed using ImageJ software. The antibodies used are listed below: TINAGL1 (1 : 1000, Proteintech Cat# 12077-1-AP, RRID: AB_2058942), HCV Core (1 : 1000, Abcam Cat# ab2740, RRID: AB_303265), α-SMA (1 : 1000, Abcam Cat# ab7817, RRID: AB_262054), TIMP1 (1 : 500, Santa Cruz Biotechnology Cat# sc-365905, RRID: AB_10848565), COL1A1 (1 : 1000, Cell Signaling Technology Cat# 84336, RRID:AB_2800036), PDGFRβ (1 : 1000, Cell Signaling Technology Cat# 3169 (also 3169S, 3169P), RRID: AB_2162497), PDGF-BB (1 : 1000, Abcam Cat# ab178409), Flag (1 : 5000, Proteintech Cat# 66008-4-Ig, RRID:AB_2918475), HA (1 : 1000, Cell Signaling Technology Cat# 3724 (also 3724S), RRID: AB_1549585). All primary antibodies are available in **Table S5**.

### Enzyme-linked immunosorbent assay (ELISA)

The human TINAGL1 ELISA kit (Boster, EK1766) was used to examine TINAGL1 concentration in culture medium and in cell lysates of Huh7.5 cells. The human PDGF-BB ELISA kit (KE00161, Proteintech) and mouse PDGF-BB ELISA kit (KE10034, Proteintech) were used to examine the PDGF-BB concentration in the culture medium of Huh7.5 cells and in mouse serum, respectively. The analyses were performed according to the instructions of the kit.

### Plasmid construction

The full-length and Flag-tagged cDNA of human *TINAGL1* was cloned into pcDNA3.1+ vector. The full-length human *PDGFB* cDNA with HA-tag was purchased from Sino Biological, Inc (HG10572-CY, Beijing, China). All constructs were verified by DNA sequencing analysis. All primers for plasmid are available in **Table S6**.

### Statistical analysis

Data were analyzed using GraphPad Prism 8. Data are presented as mean ± standard deviation (SD). Data were compared using an unpaired t-test, one-way ANOVA, or two-way ANOVA, as indicated in the figure legends. *P*-values of < 0.05 were considered significant.

## Results

### TINAGL1 is increased in HCV-infected patients, hepatocytes and in the liver of mice and patients with liver fibrosis

**Figure 1:**
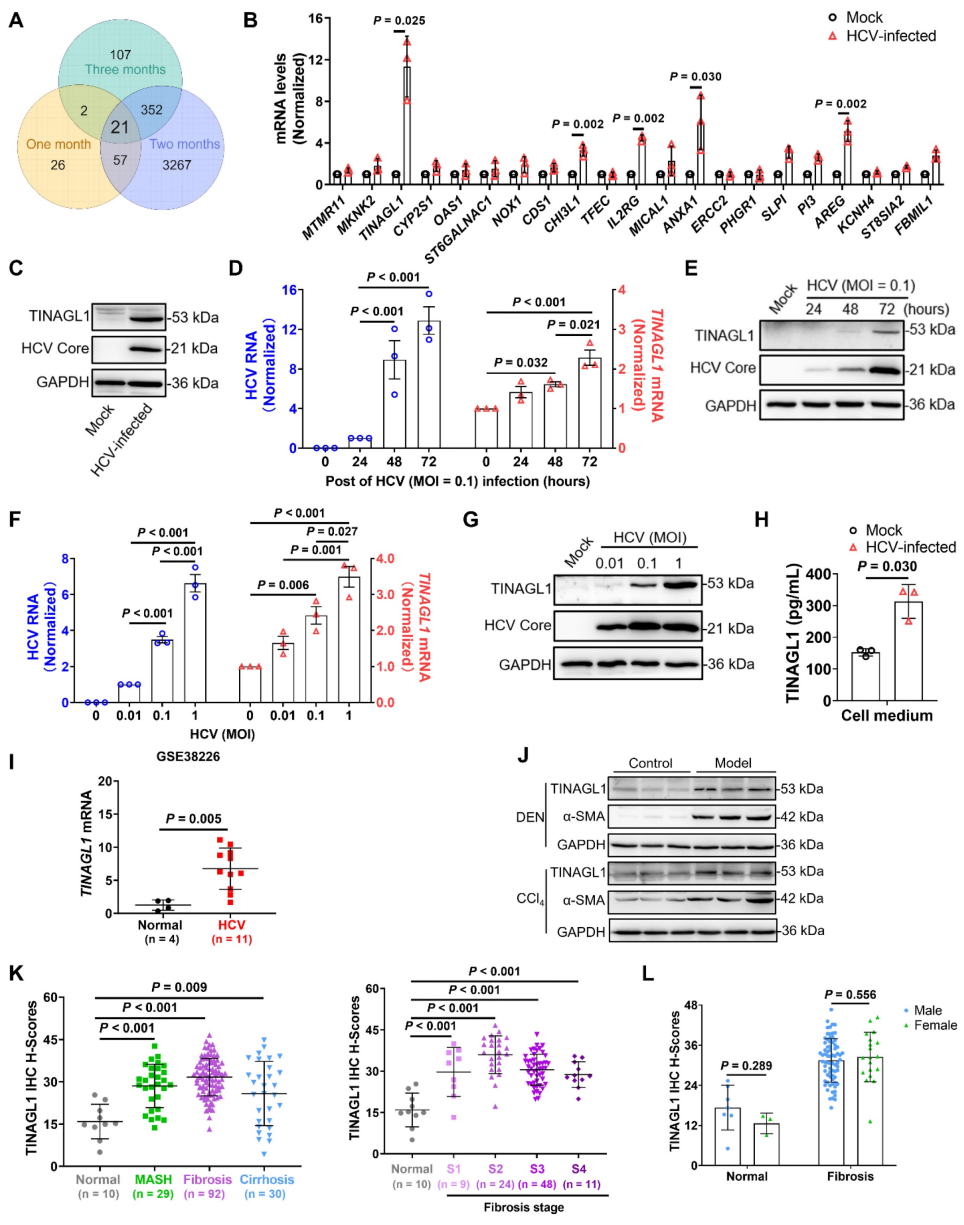
** TINAGL1 is increased in HCV-infected patients, hepatocytes and in the liver of mice and patients with fibrosis. (A)** Venn diagram analysis of genes in HCV-infected Huh7.5 cells at the indicated time points (Fold change ≥ 2). **(B-C)** RNA levels quantified by qRT-PCR **(B)** and proteins detected by Western blot **(C)** in Huh7.5 cells infected with HCV for over three months (*n* = 3).** (D-E)** RNA** (D)** and protein **(E)** levels in Huh7.5 cells infected with HCV (MOI = 0.1) for 24, 48, and 72 hours (*n* = 3). **(F-G)** RNA** (F)** and protein **(G)** levels in Huh7.5 cells infected with HCV (MOI = 0.01, 0.1, and 1) for 72 hours (*n* = 3).** (H)** TINAGL1 quantified by ELISA in the culture medium of HCV-infected Huh7.5 cells (*n* = 3). **(I)** *TINAGL1* mRNA in liver biopsies from Gene Expression Omnibus database (GSE38226).** (J)** TINAGL1 measured by Western blot in mouse livers with fibrosis. **(K-L)** Statistical summary of TINAGL1 expression in a human liver tissue array from patients with MASH (*n* = 29), fibrosis (*n* = 92), cirrhosis (*n* = 30), and normal donors (*n* = 10). Data were expressed as mean ± standard deviation. *P* values were calculated by an unpaired two-tailed Student's t-test **(B, H, I, and L)** or one-way ANOVA **(D, F, and K)** using Tukey's multiple comparisons test. α-SMA, alpha-smooth muscle actin; CCl_4_, carbon tetrachloride; DEN, diethylnitrosamine; GAPDH, glyceraldehyde-3-phosphate dehydrogenase; HCV, hepatitis C virus; TINAGL1, tubulointerstitial nephritis antigen-like 1.

To investigate the crucial factors for hepatic fibrogenesis induced by HCV infection, we used Huh7.5 cells, a subline of the human hepatoma cell line Huh7, which are easily infected with HCV due to a defect in innate antiviral signaling 35. We infected them with HCV for one to three months to analyze RNA levels by mRNA Microarray. After one, two, and three months of HCV infection, 106, 3697, and 482 genes were upregulated, respectively, compared with the uninfected cells, with 21 consensus genes upregulated (**Figure 1A** and **Table S7**). Among them, *TINAGL1*, *CHI3L1*, *IL2RG*, *AREG*, and *ANXA1* were validated at the mRNA level quantified by qRT-PCR (**Figure 1B**), and CHI3L1 36 and IL2RG 37 have been reported to be strongly associated with HCV infection, while ANXA1 has a protective effect in metabolic dysfunction-associated steatohepatitis in mice 38. In the search for more effective factors for the response to HCV-induced hepatic fibrogenesis, our attention was drawn to TINAGL1. We found that intracellular TINAGL1 was increased at the protein level after a long time of HCV infection (**Figure 1C**), and it was increased both in a time-dependent manner (**Figure 1D**, **E**) and depending on the infectious dose (**Figure 1F**, **G**) in Huh7.5 cells infected with HCV. As a secretory protein 21, TINAGL1 was also increased in the cell culture supernatants (**Figure 1H**).

To evaluate whether TINAGL1 expression is associated with HCV infection in patients, we analyzed the expression of TINAGL1 in HCV-infected patients utilizing the publicly available Gene Expression Omnibus (GEO) database from the National Center for Biotechnology Information. Human *TINAGL1* mRNA level was significantly increased in HCV-infected patients compared to healthy controls (**Figure 1I**). These results suggest that the expression of TINAGL1 is enhanced by HCV infection. The mRNA and protein levels of TINAGL1 showed no significant change in HBV gene stably transfected Hep G2.2.15 cells compared with Hep G2 cells. Meanwhile, TINAGL1 mRNA levels were slightly decreased in HBV-infected patients compared to healthy controls (GSE38941), although there was a significant difference between the mean levels of the two groups (7.87 ± 0.21 in controls and 7.72 ± 0.15 in HBV-infected patients) (**Figure S1A**, **B**).

However, we found that TINAGL1 does not affect HCV replication. The levels of HCV RNA and Core protein in HCV-infected Huh7.5 cells did not change after transfection with the TINAGL1 plasmid (**Figure S1C-E**) or TINAGL1 siRNA (**Figure S1F**, **G**), indicating that TINAGL1 in hepatocytes does not affect HCV replication. In addition, overexpression of TINAGL1 in Huh7.5 cells had no effect on the expression of HCV-associated inflammatory factors (**Figure S1H**).

To evaluate whether the expression of TINAGL1 was related to liver fibrosis, we detected the expression of TINAGL1 *in vivo*. The results showed that the expression of TINAGL1 was markedly increased in the liver of mouse models with liver fibrosis induced by diethylnitrosamine (DEN) and carbon tetrachloride (CCl_4_) (**Figure 1J**), which histopathology was shown in our previous references 32. However, we found that the change of TINAGL1 was higher with HCV infection (3- to 5-fold) than with CCl_4_ treatment (1.5- to 2-fold), hinting that TINAGL1 plays a greater role in HCV-infected patients with liver fibrosis than in patients with fibrosis caused by other factors.

Furthermore, TINAGL1 was significantly increased in the livers of patients with metabolic dysfunction associated fatty liver disease (MAFLD), especially with liver fibrosis, as shown by immunohistochemistry (IHC) staining (**Figure 1K, Figure S1I** and **Table S1**). However, the expression did not increase further with the advancing fibrosis stage (**Figure 1K**), and no significant gender differences in the expression of TINAGL1 were observed in the patients with liver fibrosis (**Figure 1L**). These results suggest that the expression of TINAGL1 is persistently increased in the livers of MAFLD patients and may contribute to the pathogenesis of MAFLD.

### TINAGL1 expression remains higher in hepatocytes after HCV elimination by high-efficiency treatment with DAAs

**Figure 2:**
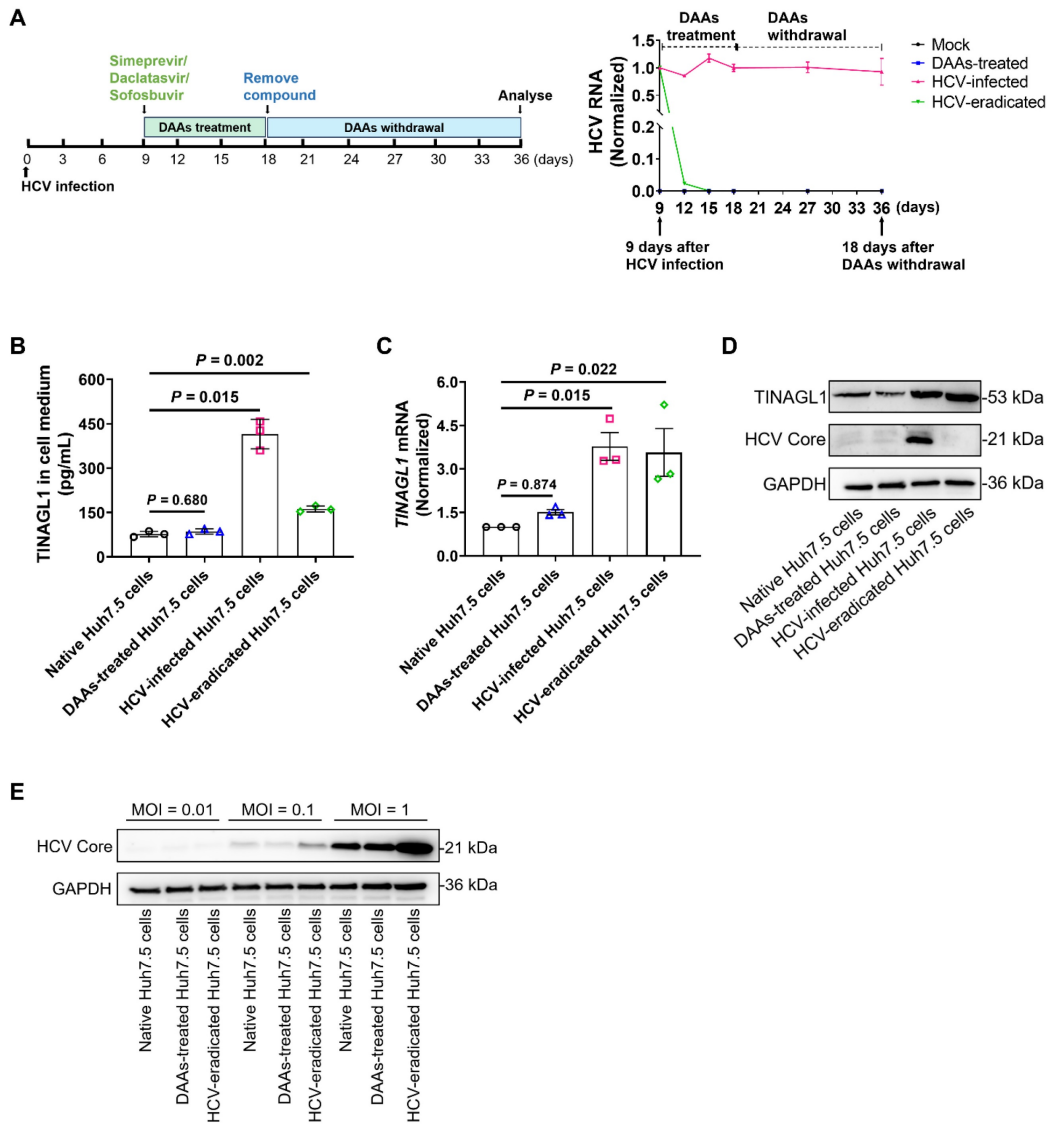
**TINAGL1 expression remains higher in hepatocytes after HCV elimination by high-efficiency treatment with DAAs**. **(A)** Schematic diagram (left) for the establishment of HCV-eradicated Huh7.5 cells and HCV RNA (right) in the cells (*n* = 3). **(B)** TINAGL1 quantified by ELISA in the culture medium of HCV-eradicated Huh7.5 cells (*n* = 3).** (C-D)** mRNA** (C)** and protein **(D)** of TINAGL1 in HCV-eradicated Huh7.5 cells (*n* = 3).** (E)** Protein levels in HCV-eradicated Huh7.5 cells after re-infection with HCV for 72 hours (*n* = 3). Data were expressed as mean ± standard deviation. *P* values were calculated by one-way ANOVA **(B, C)** using Tukey's multiple comparisons test. DAAs, direct-acting antivirals.

Studies have shown that fibrogenic signaling persists in HCV-infected patients treated with DAAs even after sustained SVR 7. Therefore, we explored whether the increased expression of TINAGL1 in hepatocytes persists after virological cure. We established an HCV-eradicated hepatocyte model using Huh7.5 cells treated with the fixed-dose combination of simeprevir, daclatasvir, and sofosbuvir (**Figure 2A**). Our results showed that secretory TINAGL1 was increased in the culture medium of HCV-eradicated Huh7.5 cells (**Figure 2B**). Accordingly, intracellular TINAGL1 expression at mRNA and protein levels was higher in HCV-eradicated Huh7.5 cells than in native control Huh7.5 cells (**Figure 2C**, **D**), indicating that the expression of TINAGL1 is still high even after HCV elimination, which is not due to drug treatment with DAAs (**Figure 2B-D**). Of note, our results showed that the HCV-eradicated Huh7.5 cells were more susceptible to reinfection with HCV compared with the native Huh7.5 cells (**Figure 2E**), suggesting that patients cured by treatment with DAAs may have a higher risk of HCV reinfection, which is consistent with clinical reports 39, 40. These results suggest that the HCV-infected hepatocytes treated with DAAs may develop more advanced lesions including fibrosis and that persistent expression of TINAGL1 may be a pathogenic factor for fibrosis in patients who have achieved a sustained SVR.

### TINAGL1 promotes the activation of hepatic stellate cells *in vitro*

**Figure 3:**
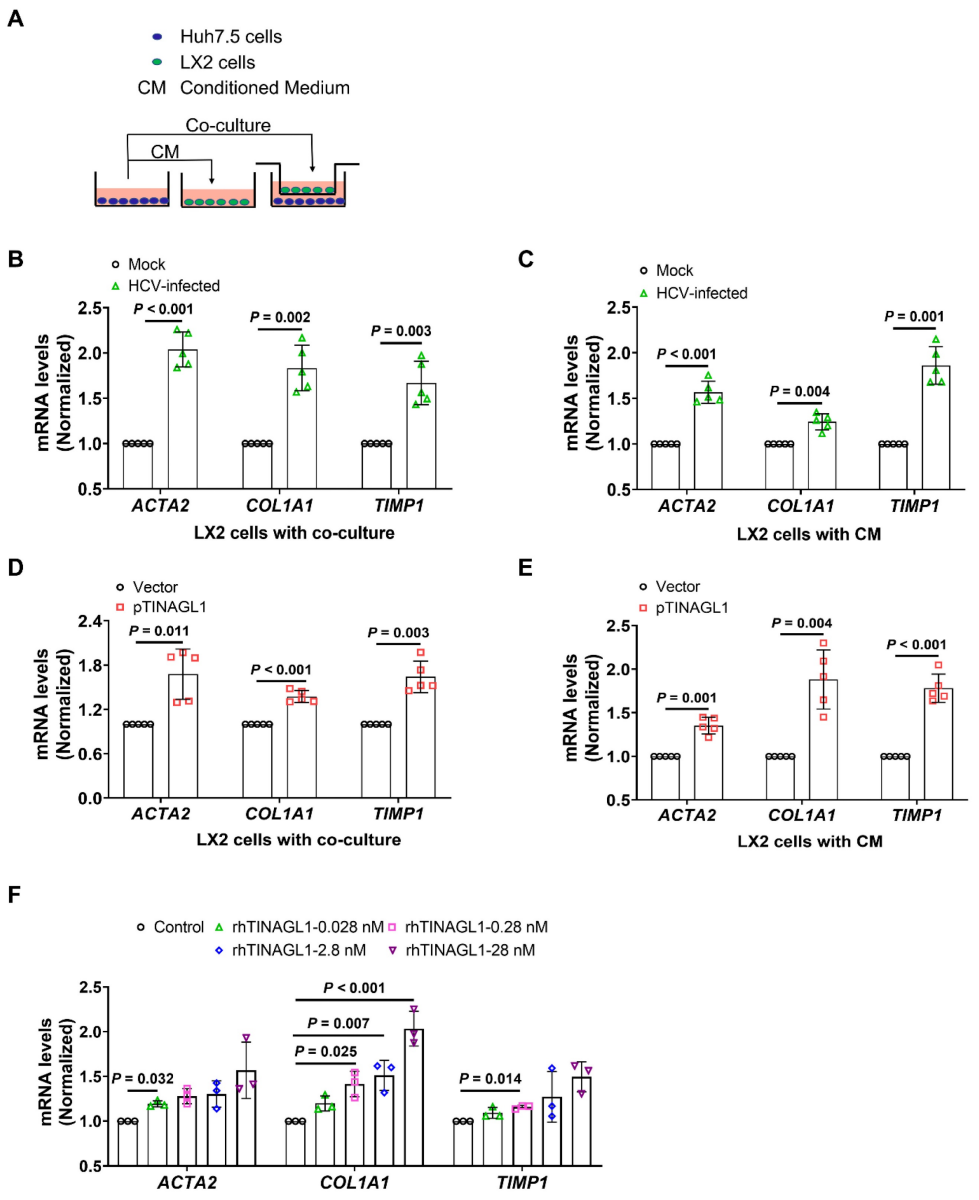
** TINAGL1 promotes the activation of hepatic stellate cells *in vitro.* (A)** Schematic diagram of the transwell insert mono- and co-culture models.** (B-C)** mRNA levels in LX-2 cells co-cultured with HCV-infected Huh7.5 cells** (B)** or their CM** (C)** (*n* = 5). **(D-E)** mRNA levels in LX-2 cells co-cultured with Huh7.5 cells transfected with the TINAGL1 plasmid** (D)** or their CM **(E)** (*n* = 5). **(F)** mRNA levels in LX-2 cells treated with rhTINAGL1 for 48 hours (*n* = 3). Data were expressed as mean ± standard deviation. *P* values were calculated by an unpaired two-tailed Student's t-test** (B-E)** or one-way ANOVA **(F)** using Tukey's multiple comparisons test. *ACTA2* (α-SMA), alpha-smooth muscle actin; CM conditioned medium; *COL1A1*, collagen type I alpha 1; rhTINAGL1, recombinant human tubulointerstitial nephritis antigen-like 1; *TIMP1*, tissue inhibitor of matrix metalloproteinase 1.

Activation of hepatic stellate cells (HSCs) is a critical event for fibrogenesis 15. Under physiological conditions, HSCs are quiescent in the Disse space, whereas they are activated by injured hepatocytes 41. Therefore, we explored whether TINAGL1 in hepatocytes activates HSCs. A crosstalk between hepatocytes and HSCs was evaluated using a conditioned medium (CM) and transwell co-culture system (**Figure 3A**). We found that co-culture with HCV-infected Huh7.5 cells significantly increased the mRNA levels of fibrogenic factors alpha-smooth muscle actin (α-SMA) (*ACTA2*), collagen type I alpha 1 (*COL1A1*), and tissue inhibitor of matrix metalloproteinase 1 (*TIMP1*), which are markers of activated HSCs, in the LX-2 cells (**Figure 3B**), suggesting that the LX-2 cells were activated. Similarly, LX-2 cells were also activated by the conditioned medium of HCV-infected Huh7.5 cells (**Figure 3C**), whereas co-culture with native Huh7.5 cells or their CM did not activate HSCs (**Figure S2A**, **B**). However, LX-2 cells were activated by co-culture with Huh7.5 cells overexpressing TINAGL1 and their CM (**Figure 3D**, **E**). Of note, the recombinant human TINAGL1 protein directly activated LX-2 cells in a concentration-dependent manner (**Figure 3F**). However, overexpression of TINAGL1 in Huh7.5 cells had no effect on the expression of TGF-β1 in LX-2 cells (**Figure S2C, D**). We do not strongly exclude the possibility that TINAGL1 affects downstream factors of the TGF signaling pathway, which remains to be further clarified. These results suggest that secretory TINAGL1 is the sponsor for the activation of LX-2 cells.

### TINAGL1 activates HSCs by stabilizing PDGF-BB

**Figure 4:**
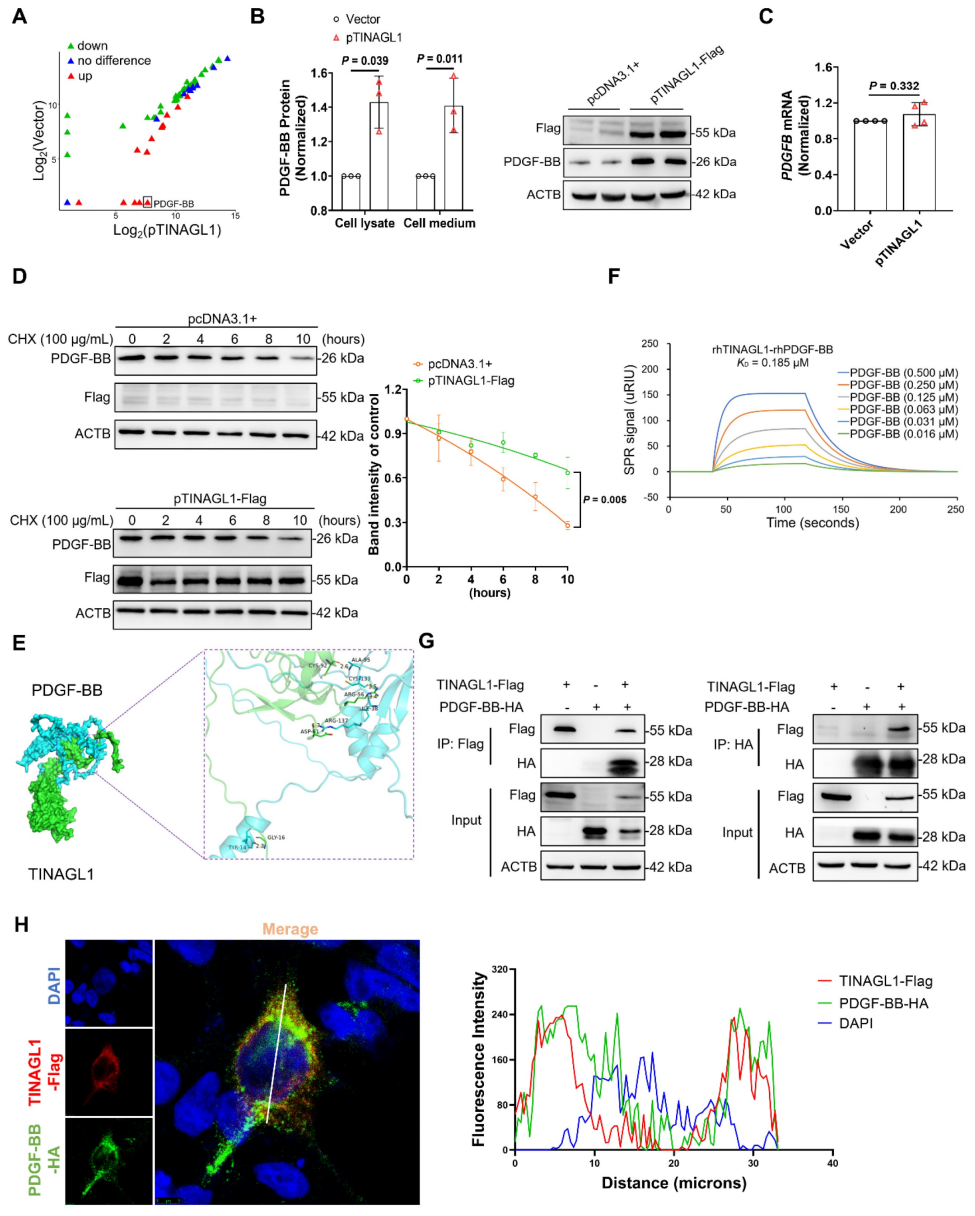
** TINAGL1 activates HSCs by stabilizing PDGF-BB. (A)** Scatter Chart of fibrosis-associated cytokines in the culture medium of Huh7.5 cells transfected with the TINAGL1 plasmid detected by a cytokine microarray assay.** (B)** PDGF-BB concentration quantified by ELISA in cell lysate and culture medium and by Western Blot in cell lysate of Huh7.5 cells transfected with the TINAGL1 plasmid (*n* = 3). **(C)** *PDGFB* mRNA level in Huh7.5 cells transfected with the TINAGL1 plasmid (*n* = 4).** (D)** Protein levels in Huh7.5 cells transfected with the *TINAGL1-Flag* plasmid and treated with cycloheximide (CHX), the intensity of protein was scanned by Image J (*n* =3). **(E)** Binding mode between human TINAGL1 and PDGF-BB. **(F)** Binding affinity between rhTINAGL1 (C-6 × His) and rhPDGF-BB proteins detected by SPR method. **(G)** Interaction of TINAGL1 and PDGF-BB in HEK293T cells detected by co-immunoprecipitation. **(H)** Co-localization of TINAGL1 (red) and PDGF-BB (green) in HEK293T cells (Scale bar: 5 μm). Nuclear (blue). Statistical analysis of co-localization of TINAGL1 and PDGF-BB fluorescence intensities was performed using ImageJ software. Data were expressed as mean ± standard deviation. *P* values were calculated by an unpaired two-tailed Student's t-test **(B-D).** ACTB, beta-actin; CHX, cycloheximide; PDGF-BB, platelet-derived growth factor BB; rhPDGF-BB, recombinant human platelet-derived growth factor BB; rhTINAGL1, recombinant human tubulointerstitial nephritis antigen-like 1; SPR, surface plasmon resonance.

To investigate how TINAGL1 activates HSCs, we identify potential profibrotic factors in the culture medium of Huh7.5 cells transfected with the TINAGL1 plasmid using a fibrosis-based human cytokine array technology. Four proteins, including glial cell-derived neurotrophic factor, leukemia inhibitory factor, brain-derived neurotrophic factor, and platelet-derived growth factor-BB (PDGF-BB), were significantly increased (**Figure 4A** and **Table S2**), with PDGF-BB showing the highest increase of over 100-fold. It is reported that liver-specific overexpression of PDGF-BB triggers the activation of HSCs 42, suggesting that the activation of HSCs by TINAGL1 may be associated with PDGF-BB.

Therefore, we analyzed the relationship between TINAGL1 and PDGF-BB. We found that PDGF-BB was increased in the supernatants and cell lysates of Huh7.5 cells transfected with the TINAGL1 plasmid (**Figure 4B**), whereas the mRNA level of PDGF-BB in the cells did not alter with the transfection of the TINAGL1 plasmid (**Figure 4C**), suggesting that TINAGL1 may affect PDGF-BB expression at the post-transcriptional level. Indeed, overexpression of TINAGL1 prolonged the half-life of PDGF-BB **(Figure 4D)**, suggesting that TINAGL1 stabilizes PDGF-BB. After searching for potential interactions in the GeneMANIA database, we found that there is a direct interaction between TINAGL1 and PDGFB (**Figure S3A**), which was confirmed using Discovery Studio 4.5 software and characterized by visible hydrogen bonds and hydrophobic interactions with a strong binding energy of - 8.04 kcal/mol (**Figure 4E**). The binding affinity between TINAGL1 and PDGF-BB was further validated by a surface plasmon resonance (SPR) assay, with a *K*_D_ value of 0.185 μM (**Figure 4F**). After co-immunoprecipitation, TINAGL1 was found to physically interact with PDGF-BB in HEK293 cells (**Figure 4G**) and Huh7.5 cells (**Figure S3B**). The co-localization assay by immunofluorescence confocal microscopy showed that TINAGL1 and PDGF-BB can be co-localized in the cytoplasm of HEK293T cells (**Figure 4H**). These data suggest that TINAGL1 directly interacts with PDGF-BB and thus stabilizes PDGF-BB. However, the manner in which TINAGL1 stabilizes PDGF-BB requires further investigation.

**Figure 5:**
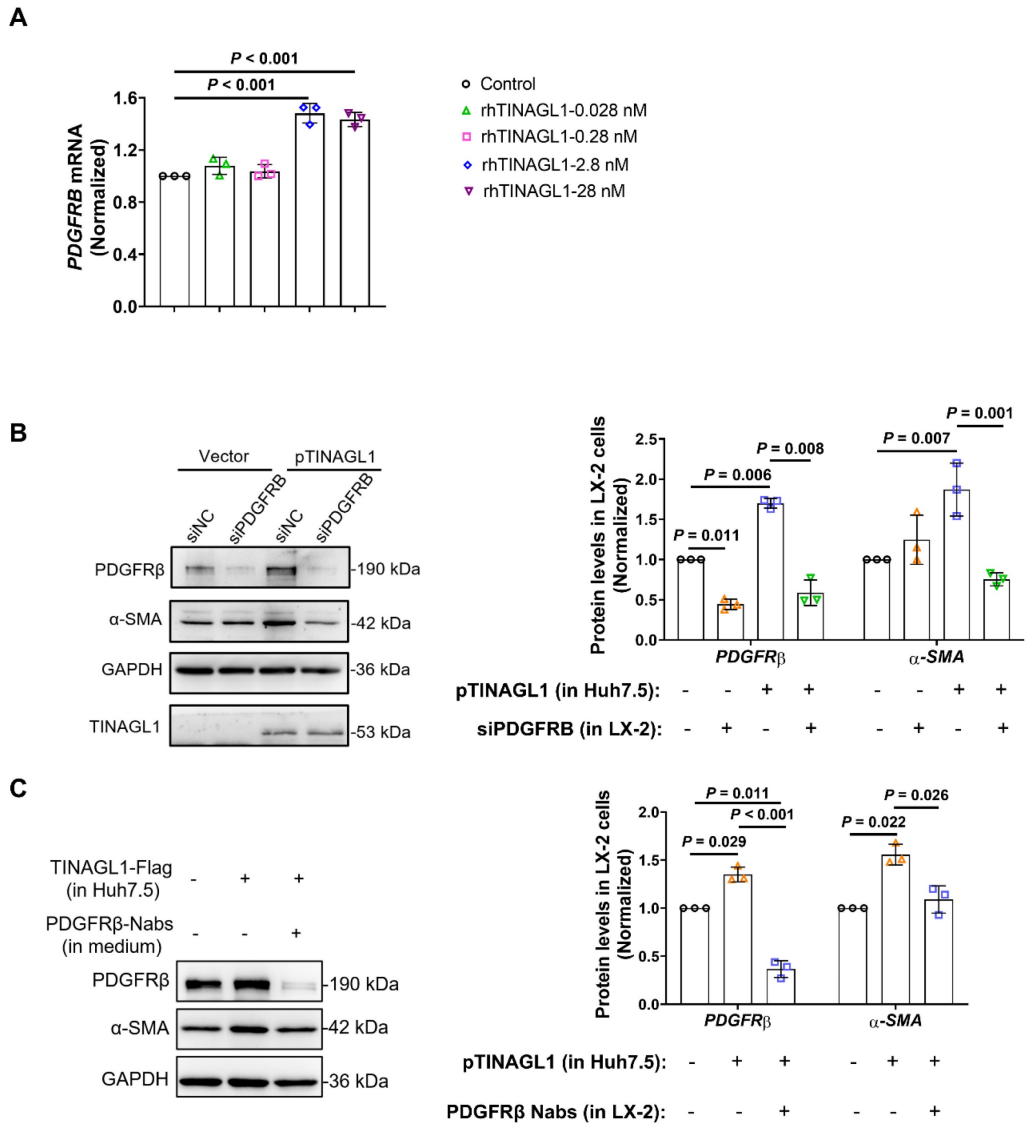
** TINAGL1 activates HSCs by PDGF-BB/PDGFRβ pathway. (A)** *PDGFRB* mRNA levels in LX-2 cells treated with rhTINAGL1 for 48 hours (*n* = 3). **(B-C)** Protein levels of α-SMA and PDGFRβ in LX-2 cell and TINAGL1 in cell medium after co-culture with Huh7.5 cells transfected with TINAGL1 plasmid, siRNA **(B)** or neutralizing antibody** (C)** against PDGFRβ, the intensity of protein was scanned by Image J (*n* =3). Data were expressed as mean ± standard deviation. *P* values were calculated by one-way ANOVA **(A-C)** using Tukey's multiple comparisons test. α-SMA, alpha-smooth muscle actin; PDGFRβ, platelet-derived growth factor receptor beta; rhTINAGL1, recombinant human tubulointerstitial nephritis antigen-like 1.

We then examined whether the activation of HSCs by TINAGL1 is through PDGF-BB. Platelet-derived growth factor receptor beta (PDGFRβ), the receptor of PDGF-BB, is a key mediator of liver injury and fibrogenesis *in vivo* and contributes to the poor prognosis of HCV-related cirrhosis 43. We found that recombinant human TINAGL1 activates LX-2 cells through the activation of PDGFRβ (**Figure 5A**). After co-culture with Huh7.5 cells overexpressing TINAGL1 by transfection of the TINAGL1 plasmid, the levels of α-SMA and PDGFRβ were significantly upregulated in LX-2 cells (**Figure 5B**, **C**). However, the activation of LX-2 cells by TINAGL1 was downregulated by specific siRNA (**Figure 5B**) and neutralizing antibody against PDGFRβ (**Figure 5C**), suggesting that activation of HSCs by TINAGL1 is associated with the PDGF-BB/PDGFRβ pathway.

### Liver-specific overexpression of TINAGL1 initiates and exacerbates liver fibrosis in mice

**Figure 6:**
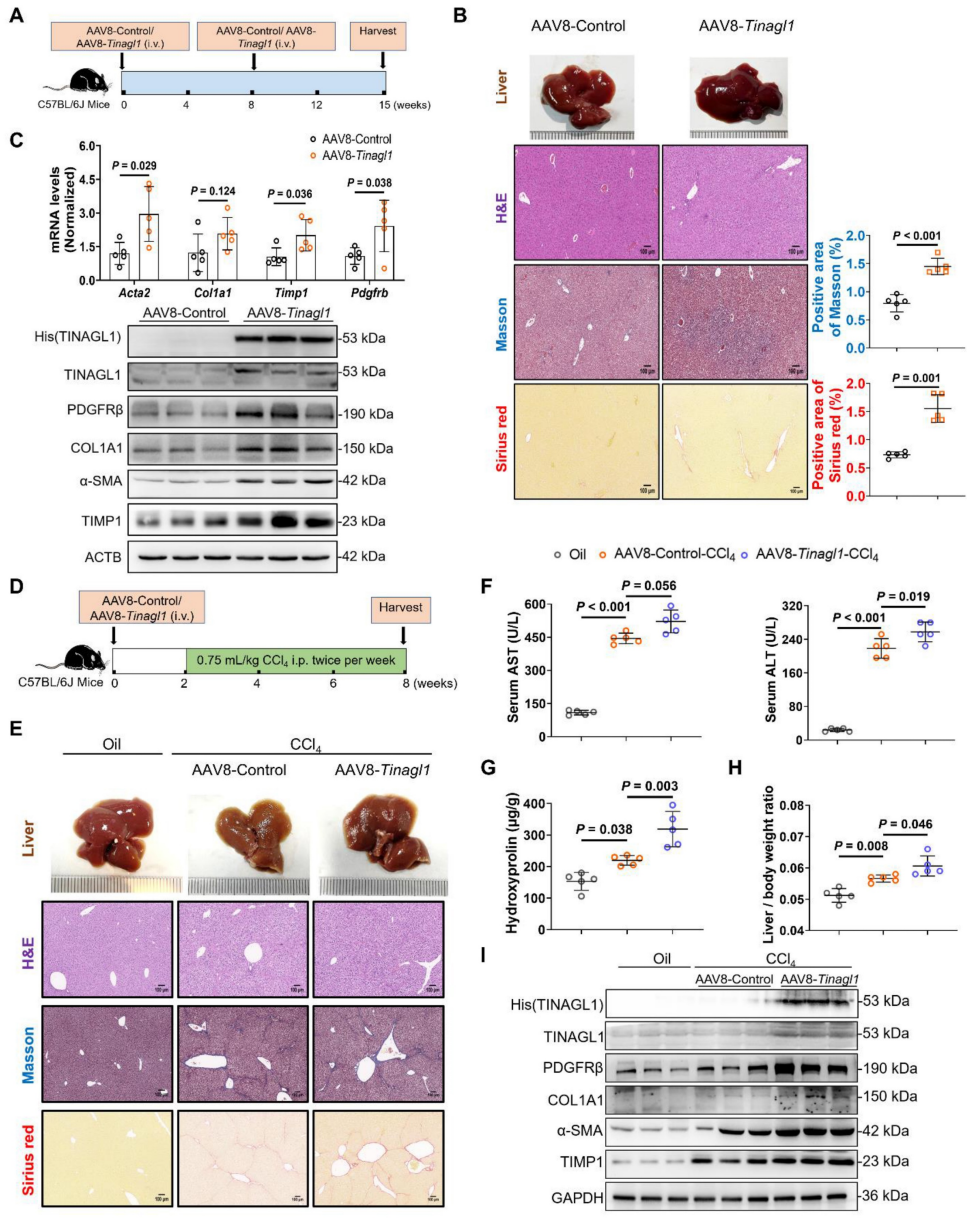
** Liver-specific overexpression of TINAGL1 initiates and exacerbates liver fibrosis in mice. (A)** Schematic overview of the experimental setup for **(B-C)**. **(B)** H&E staining (Scale bar: 100 μm), Masson staining (Scale bar: 100 μm), and Sirius red staining (Scale bar: 100 μm) of mouse livers. Quantification of Masson and Sirius red staining with Image J. **(C)** mRNA and protein levels in mouse livers (*n* = 5).** (D)** Schematic overview of the experimental setup for (E-I). **(E)** H&E staining (Scale bar: 100 μm), Masson staining (Scale bar: 100 μm), and Sirius red staining (Scale bar: 100 μm) of mouse livers. **(F)** AST and ALT in the serum of mice (*n* = 5). **(G)** Hydroxyproline content in mouse livers (*n* = 5). **(H)** Liver / body weight ratio (*n* = 5). **(I)** Protein levels in mouse livers. Data were expressed as mean ± standard deviation. *P* values were calculated by an unpaired two-tailed Student's t-test **(B-C)** or one-way ANOVA** (F-H)** using Tukey's multiple comparisons test. *Acta2* (α-SMA), alpha-smooth muscle actin; ALT, alanine aminotransferase; AST, aspartate aminotransferase; CCl_4_, carbon tetrachloride; COL1A1, collagen type I alpha 1; PDGFRβ, platelet-derived growth factor receptor beta; TIMP1, tissue inhibitor of matrix metalloproteinase 1.

Next, we investigated the profibrotic function of TINAGL1 *in vivo*. We used adeno-associated virus 8 (AAV8) to specifically overexpress TINAGL1-His (AAV8-*Tinagl1*) in the liver of C57BL/6J mice by intravenous injection into the tail (**Figure 6A**). TINAGL1-His was specifically maintained in the liver for 15 weeks (**Figure S4A**, **B**). Higher expression of TINAGL1 had no effect on mouse body weight (**Figure S4C**), liver / body weight ratio (**Figure S4D**), and serum triglycerides (**Figure S4E**), but resulted in significant histologic injury (**Figure 6B**), which was accompanied by a significant increase in serum aspartate aminotransferase (AST) and alanine aminotransferase (ALT) (**Figure S4F**). The mRNA and protein levels of fibrogenesis-related factors, such as α-SMA (Acta2), COL1A1, TIMP1, and PDGFRβ, were significantly increased in the liver of AAV8-*Tinagl1* mice (**Figure 6C**). Meanwhile, proinflammatory cytokines TNFα, IL6, and IL-1β were also increased (**Figure S4G**). However, there was no difference in TGF-β1 expression in the liver (**Figure S4H**), which is consistent with the results in the co-culture cells (**Figure S2D**). Of note, the serum level of PDGF-BB was slightly increased in the AAV8-*Tinagl1* mice (**Figure S4I**), whereas the PDGF-BB mRNA level did not change (**Figure S4J**). However, the Oil Red O (ORO) staining results showed that overexpression of TINAGL1 in the mice could not develop liver steatosis during the treatment period (**Figure S4K**). These results indicate that a higher level of *Tinagl1* in the liver initiates mouse liver fibrosis.

C57BL/6J mice were injected intravenously with AAV8-*Tinagl1*. After two weeks, the mice were injected intraperitoneally with CCl_4_ twice a week for six weeks (**Figure 6D** and **Figure S5A**). As expected, injection of CCl_4_ resulted in liver fibrosis, showing significant liver pathological findings with Masson staining and Sirius red staining (**Figure 6E**) and liver injury with higher serum AST and ALT levels (**Figure 5F**) compared to the oil control group. Overexpression of TINAGL1 exacerbated the progression of liver fibrosis, showing higher positive areas of Masson and Sirius red (**Figure 6E** and **Figure S5B**), higher serum levels of AST and ALT (**Figure 6F**), and higher content of hydroxyproline (**Figure 6G**) compared to the AAV8-Control group, in parallel with a significant increase in liver / body weight ratio (**Figure 6H**), without affecting body weight (**Figure S5C**). Of note, overexpression of TINAGL1 further increased fibrogenesis-related factors, proinflammatory cytokines, and PDGFRβ in the liver at the mRNA and protein levels (**Figure 6I** and **Figure S5D**). Serum PDGF-BB level was further increased by the overexpression of TINAGL1 compared with the control groups (**Figure S5E**), which is consistent with the results of AAV-*Tinagl1* mice (**Figure S4I**). Consistently, overexpression of TINAGL1 in mice could not develop liver steatosis in CCl_4_-induced mice (**Figure S5F**). These results demonstrated that higher TINAGL1 in the liver exacerbates the progression of liver fibrosis through the PDGF-BB/PDGFRβ signaling pathway.

### HCV-eradicated Huh7.5 cells treated with DAAs promote tumorigenesis in mice

**Figure 7:**
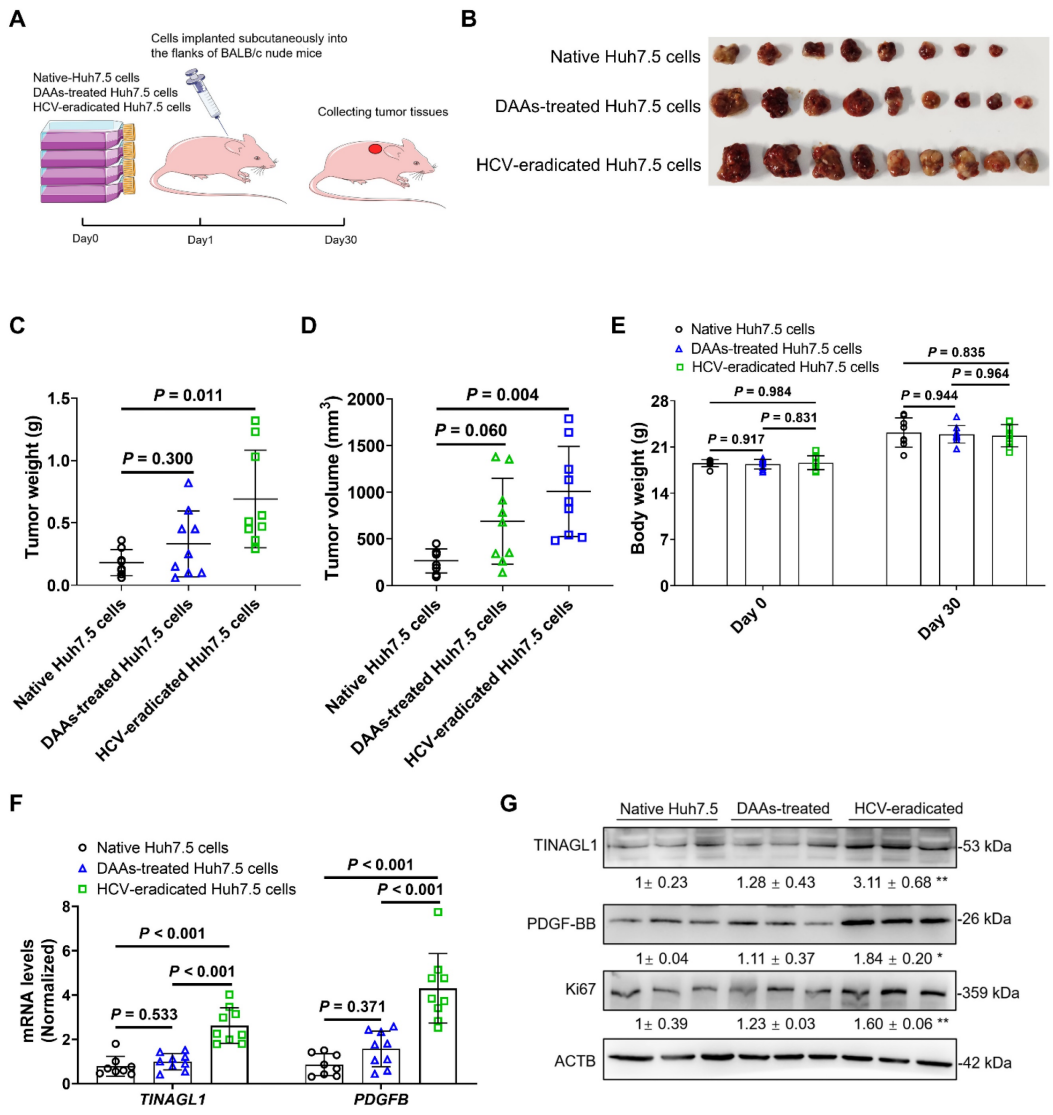
** HCV-eradicated Huh7.5 cells treated with DAAs promote tumorigenesis in mice. (A)** The process of constructing subcutaneous xenograft tumor in BALB/c nude mice. **(B-E)** Tumor tissue mass **(B)**, tumor weight **(C)**, tumor volume** (D)**, and body weight** (E)** (*n* = 8 or 9). **(F-G)** mRNA** (F)** and protein** (G)** levels in tumors (*n* = 8 or 9). Data were expressed as mean ± standard deviation. *P* values were calculated by one-way ANOVA **(C-F)** using Tukey's multiple comparisons test. DAAs, direct-acting antivirals; HCV, hepatitis C virus.

HCV-infected patients are still at risk for HCC even after virological cure by treatment with DAAs 9. Some studies have reported that HCV-expressing cell lines enhance tumorigenicity 44, 45. In this study, we investigated the tumorigenicity of HCV-eradicated Huh7.5 cells in BALB/c nude mice using the subcutaneous xenograft method (**Figure 7A**). The mass, weight, and volume of tumor tissues in the xenograft mice (**Figure 7B-D**) arising from HCV-eradicated Huh7.5 cells were significantly larger compared to the control Huh7.5 cells, with no effect on body weight (**Figure 7E**), which is consistent with clinical outcomes 46-48. The tumorigenicity of Huh7.5 cells did not alter as a result of long-term treatment with DAAs (**Figure 7B-D**), agreeing with the safety of the combination treatment of DAAs 49. The mRNA and protein levels of TINAGL1 and PDGF-BB were increased in the tumor tissues arising from HCV-eradicated Huh7.5 cells (**Figure 7F**, **G**), in parallel with an increase of the tumor proliferative marker Ki67 (**Figure 7G**). These results suggest that increased expression of TINAGL1 and PDGF-BB may be associated with tumorigenesis of HCV-eradicated Huh7.5 cells. These results highlight that the higher TINAGL1 level is a possible cause of the risk of HCC after SVR.

### Liver-specific knockdown of TINAGL1 prevents the progression of liver fibrosis in mice induced by CCl_4_

**Figure 8:**
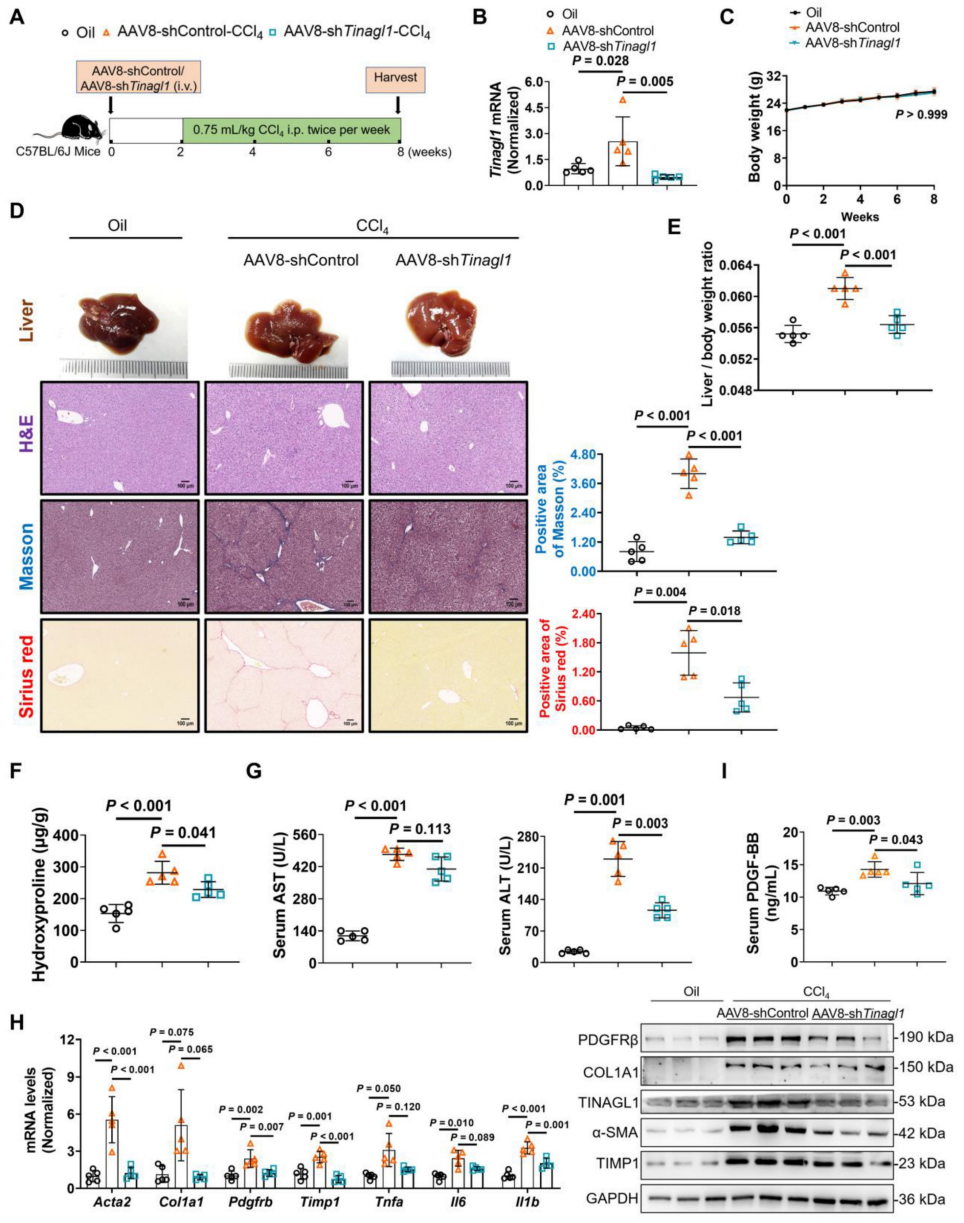
** Liver-specific knockdown of TINAGL1 prevents the progression of liver fibrosis in mice induced by CCl_4_. (A)** Schematic overview of the experimental setup. **(B)** mRNA level of *Tinagl1* in mouse livers (*n* = 5).** (C)** Body weight (*n* = 5). **(D)** H&E staining (Scale bar: 100 μm), Masson staining (Scale bar: 100 μm), and Sirius Red staining (Scale bar: 100 μm) of mouse livers. Quantification of Masson and Sirius red staining with Image J.** (E)** Liver / body weight ratio (*n* = 5). **(F)** Hydroxyproline content in mouse livers (*n* = 5).** (G)** Serum AST and ALT in mice (*n* = 5). **(H)** mRNA and protein levels in mouse livers (*n* = 5). **(I)** Serum PDGF-BB level quantified by ELISA (*n* = 5). Data were expressed as mean ± standard deviation. *P* values were calculated by one-way ANOVA **(B, D-I)** using Tukey's multiple comparisons test or two-way ANOVA **(C)** using Bonferroni's multiple comparisons test. *Acta2* (α-SMA), alpha-smooth muscle actin; AST, aspartate aminotransferase; ALT alanine aminotransferase; COL1A1, collagen type I alpha 1; PDGFRβ, platelet-derived growth factor receptor beta; TIMP1, tissue inhibitor of matrix metalloproteinase 1;* Il1b*, interleukin-1 beta; *Il6,* interleukin 6; *Tnfa*, tumor necrosis factor alpha.

Based on our evidence that TINAGL1 is a profibrotic factor for liver fibrosis, we first evaluated whether targeted silencing of TINAGL1 prevents the progression of liver fibrosis. C57BL/6J mice were injected intravenously with AAV8-sh*Tinagl1* to persistently express the shRNA for *Tinagl1*. After two weeks, the mice were injected intraperitoneally with CCl_4_ twice a week for six weeks (**Figure 8A**). As expected, AAV8-sh*Tinagl1* strongly downregulated the expression of TINAGL1 in the liver compared to the AAV8-control, although CCl_4_ increased the level of TINAGL1 (**Figure 8B**). AAV8-sh*Tinagl1* had no effect on the mouse body weight (**Figure 8C**) but significantly decreased the degree of liver fibrosis (**Figure 8D**), liver / body weight ratio (**Figure 8E**), liver hydroxyproline content (**Figure 8F**), serum ALT and AST levels **(Figure 8G)**, and also decreased fibrotic factors, proinflammatory cytokines, and PDGFRβ at mRNA and protein levels (**Figure 8H**) compared to the CCl_4_-injected group. Synchronously, serum PDGF-BB levels were also significantly decreased due to hepatocyte-specific knockdown of TINAGL1 in the mice compared with the CCl_4_-injected group (**Figure 8I**). These results suggest that inhibition of TINAGL1 has a preventive effect on the progression of liver fibrosis.

### Liver-specific knockdown of TINAGL1 alleviates liver fibrosis in mice induced by CCl_4_

**Figure 9:**
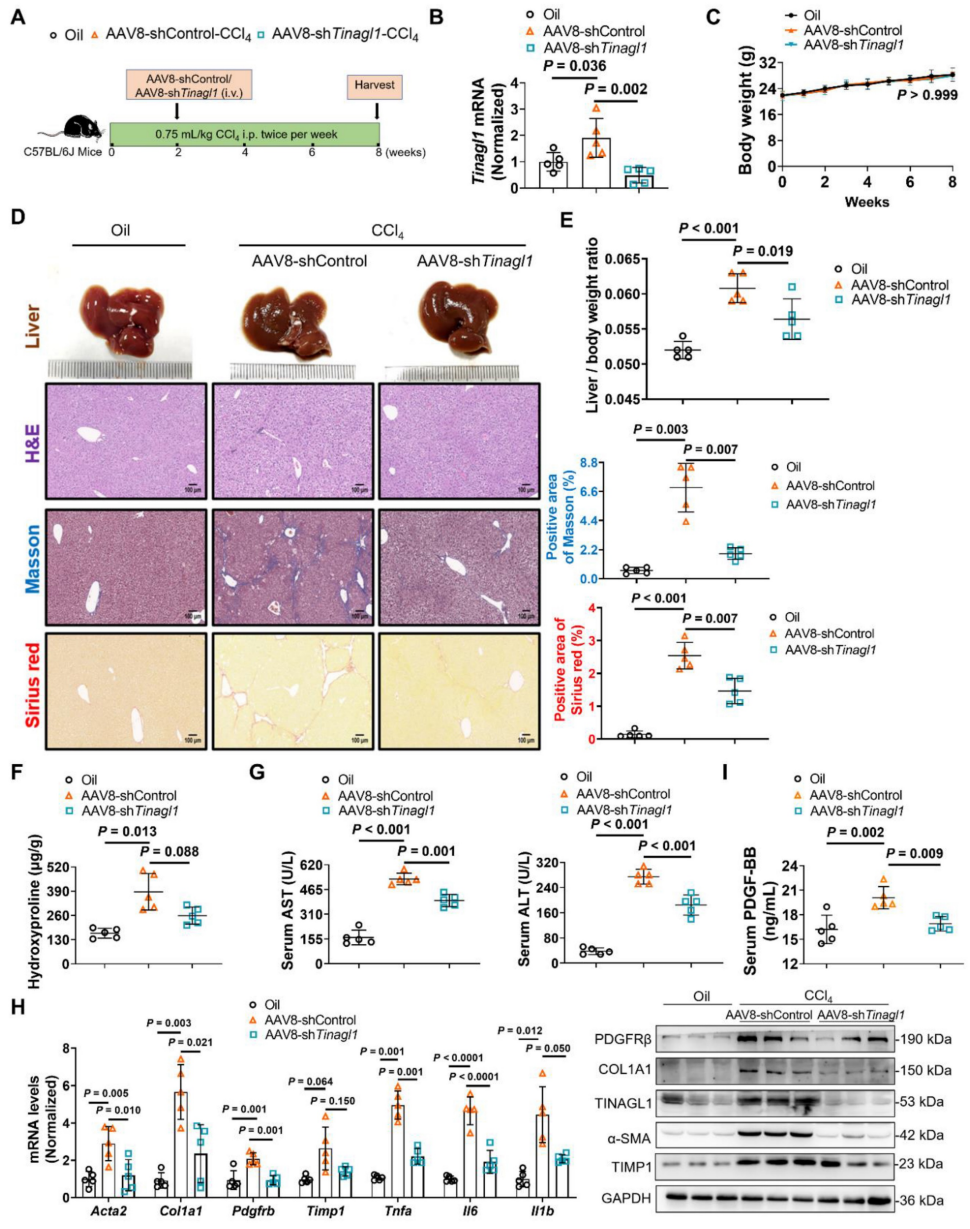
**Liver-specific knockdown of TINAGL1 alleviates liver fibrosis in mice induced by CCl_4_. (A)** Schematic overview of the experimental setup. **(B)** mRNA level of *Tinagl1* in mouse livers (*n* = 5). **(C)** Body weight (*n* = 5).** (D)** H&E staining (Scale bar: 100 μm), Masson staining (Scale bar: 100 μm), and Sirius Red staining (Scale bar: 100 μm) of mouse livers. Quantification of Masson and Sirius red staining with Image J.** (E)** Liver / body weight ratio (*n* = 5). **(F)** Hydroxyproline content in mouse livers (*n* = 5). **(G)** Serum ALT and AST in mice (*n* = 5). **(H)** mRNA and protein levels in mouse livers (*n* = 5). **(I)** Serum PDGF-BB level quantified by ELISA (*n* = 5). Data were expressed as mean ± standard deviation. *P* values were calculated by one-way ANOVA **(B, D-I)** using Tukey's multiple comparisons test or two-way ANOVA** (C)** using Bonferroni's multiple comparisons test. *Acta2* (α-SMA), alpha-smooth muscle actin; AST, aspartate aminotransferase; ALT alanine aminotransferase; COL1A1, collagen type I alpha 1; PDGFRβ, platelet-derived growth factor receptor beta; TIMP1, tissue inhibitor of matrix metalloproteinase 1;* Il1b*, interleukin-1 beta; *Il6,* interleukin 6; *Tnfa*, tumor necrosis factor alpha.

Then, we evaluated whether targeted silencing of TINAGL1 has a therapeutic effect on liver fibrosis. C57BL/6J mice were injected intraperitoneally with CCl_4_ twice a week for 8 weeks. In the second week, the mice were injected intravenously with AAV8-sh*Tinagl1* (**Figure 9A**). Similarly, AAV8-sh*Tinagl1* strongly decreased the expression of TINAGL1 in the liver compared to the AAV8-control (**Figure 9B**), with no effect on the mouse body weight (**Figure 9C**). Of note, CCl_4_-induced liver fibrosis was significantly worse at 8 weeks (**Figure 9D-H**) than at 6 weeks (**Figure 8**), while AAV8-sh*Tinagl1* significantly reduced the degree of liver fibrosis (**Figure 9D**) and the liver / body weight ratio (**Figure 9E**), significantly reduced liver hydroxyproline content (**Figure 9F**) and serum levels of ALT and AST **(Figure 9G)**, and decreased fibrotic factors, proinflammatory cytokines, and PDGFRβ at mRNA and protein levels (**Figure 9H**) compared to the CCl_4_-injected group. Meanwhile, serum PDGF-BB levels were significantly decreased by hepatocyte-specific knockdown of TINAGL1 (**Figure 9I**). Taken together, our findings suggest that inhibition of TINAGL1 may be of potential value for the treatment of liver fibrosis.

To find potential compounds targeting TINAGL1, we performed a high-throughput screening method using the human recombinant TINAGL1 protein with His-tag based on SPR technology (**Figure S6A**). A total of 1760 FDA-approved drugs and 640 natural products were screened and compounds capable of binding to TINAGL1 were preliminarily obtained (**Figure S6B**). The results showed that Lomefloxacin and Acefylline can bind directly to TINAGL1 (**Figure S6C, D**), suggesting that TINAGL1 could be an innovative therapeutic target for new drugs, although the potential antifibrotic effects of the two compounds need to be further validated.

## Discussion

It is of great clinical importance to understand the mechanism of potential factors that promote liver fibrosis even after virological cure by effective treatment with DAAs. Here, we found that TINAGL1 is significantly increased in HCV-infected and HCV-eradicated hepatocytes, as well as in the liver of mouse models and patients with liver fibrosis. Overexpression of TINAGL1 initiates and exacerbates liver fibrosis in mice. Mechanistically, TINAGL1 directly binds to and stabilizes the known profibrotic factor PDGF-BB, which leads to the activation of HSCs and triggers liver fibrosis through the PDGF-BB/PDGFRβ signaling pathway. Knockdown of TINAGL1 produces preventive and therapeutic effects on liver fibrosis in mice. Therefore, our results highlight that TINAGL1 is a new profibrotic factor in the liver, even after cure with DAA-based therapy, and that targeting TINAGL1 may be an innovative strategy for the treatment of liver fibrosis, especially in the post-antiviral era.

HCV infection is one of the most important causes of liver fibrosis 16. Although treatment with DAAs can completely eliminate HCV in patients, increasing clinical evidence suggests that patients treated with DAAs are still at a high risk of liver fibrosis, which may even develop into cirrhosis and liver cancer 9. Therefore, we seek to uncover the abnormal high-risk profibrotic factors that persist in HCV-infected hepatocytes, even in HCV-eradicated patients treated with high-efficiency DAAs. Although many factors have been reported to show aberrant expression after treatment with DAAs, their specific mechanisms and target potency have not yet been fully validated 7, 9. Our data demonstrated that TINAGL1 was significantly increased in HCV-infected hepatocytes (**Figure 1**) and highlighted the expression of TINAGL1 remains higher after HCV elimination by treatment with DAAs (**Figure 2**). However, the detailed mechanism by which HCV modulates TINAGL1 expression remains to be further clarified. TINAGL1 acts as an upstream factor of the well-known profibrotic factor PDGF-BB and promotes fibrosis by directly binding to and stabilizing PDGF-BB in hepatocytes (**Figure 4**), which is distinctly different from the classical TGF-β1 pathway, which is the most potent profibrotic factor and activates HSCs in an SMAD2- or SMAD3-dependent manner in most models 50. Furthermore, TINAGL1 is a secretory protein 21 and potential carcinoma tumorigenicity 25-27. These clues strongly suggest that TINAGL1 may serve as a potential diagnostic marker for liver fibrosis in patients. However, further clinical investigations are needed to clarify this issue, particularly in HCV-eradicated patients with a sustained SVR by combined treatment with DAAs. We also found that the expression of TINAGL1 was markedly increased in the livers of patients with MASH (**Figure S7A**). Meanwhile, we found that the mRNA and protein levels of TINAGL1 were slightly increased in Hep G2 cells treated with free-fatty acid (**Figure S7B, C**), hinting that TINAGL1 might serve as a factor in the pathogenesis of steatosis. However, these results need to be further confirmed.

Although many drugs that target the hepatic stearoyl-CoA desaturase 51, C-C chemokine receptors 2 / 5 52, 53, farnesoid X receptor 54, nicotinamide adenine dinucleotide phosphate oxidase 3 55, cyclophilin 56, fibroblast growth factor 57, galectin-3 58, glucagon-like peptide-1 59, caspase 60, and peroxisome proliferator-activated receptor gamma 61 are currently being evaluated for the treatment of liver fibrosis and some of them have shown positive clinical outcomes 51-54, 57, but no specific drug has yet been approved for use in the clinic 20. Therefore, the development of new targets and targeted drugs is an important strategy to solve this problem. In this work, we found that knockdown of TINAGL1 by the AAV8-vector has significant preventive and therapeutic effects on liver fibrosis in mice (**Figure 8-9**), highlighting the potential of TINAGL1 as an innovative therapeutic target for the development of new drugs for the treatment of liver fibrosis. Our screening results using SPR technology showed that Lomefloxacin and Acefylline can bind directly to TINAGL1 (**Figure S6**), although the potential antifibrotic effects of the two compounds need to be further validated. In the future, TINAGL1 may serve as an innovative target for the development of a series of macromolecular antibodies and small molecule inhibitors for further clinical trials.

In summary, this present study uncovered an important role for TINAGL1 in liver fibrosis after HCV elimination by DAAs. Mechanistically, TINAGL1 directly binds to and stabilizes PDGF-BB, leading to activation of HSCs and triggering liver fibrosis through the PDGF-BB/PDGFRβ signaling pathway. Our findings provide new insights into liver fibrosis after virological cure and suggest that targeting TINAGL1 may be a therapeutic potential for the treatment of liver fibrosis.

## Supplementary Material

Supplementary figures and tables.

## Abbreviations

AAV8: adeno-associated virus 8
ALT: alanine transaminase
α-SMA (ACTA2): alpha-smooth muscle actin
AST: aspartate transaminase
CCl_4_: carbon tetrachloride
COL1A1: collagen type I alpha 1
DAAs: direct-acting antivirals
DEN: diethylnitrosamine
ECM: extracellular matrix
HCC: hepatocellular carcinoma
HCV: hepatitis C virus
HSCs: hepatic stellate cells
IHC: immunohistochemistry
LSECs: liver sinusoidal endothelial cells
MAFLD: metabolic dysfunction-associated fatty liver disease
PDGF-BB: platelet-derived growth factor-BB
PDGFRβ: platelet-derived growth factor receptor beta
TG: triacylglycerol
SMAD: small mothers against decapentaplegic protein
SVR: sustained virological response
TGF-β1: transforming growth factor-beta1
TIMP1: tissue inhibitor of matrix metalloproteinase 1
TINAGL1: tubulointerstitial nephritis antigen-like 1
