# Supplementary material for: Tubulointerstitial nephritis antigen-like 1 promotes the progression of liver fibrosis after HCV eradication with direct-acting antivirals
Source: Int J Biol Sci. 2025 Jan 1;21(2):802–22. doi: 10.7150/ijbs.103305 (PMC11705640; doi:10.7150/ijbs.103305)
Supplement: Supplementary file 1 — Supplementary figures and tables. [file ijbsv21p0802s1.pdf]

Supplemental information for:

Tubulointerstitial nephritis antigen-like 1 promotes the progression of liver fibrosis after HCV eradication with direct-acting antivirals

Lei Lei [1, §], Hu Li [1, 2], Xue-Kai Wang [1], Jian-Rui Li [1, 3], Han Sun [1], Hong-Ying Li [1], Jia-Yu Li [1], Mei Tang [1], Jing-Chen Xu [1], Biao Dong [1, 2], Yue Gong [1], Dan-Qing Song [3, 4], Jian-Dong Jiang [1, 2, 3, 4], Zong-Gen Peng [1, 2, 3, 4 *]

This section file includes:

Supplemental Figure S1 to S7

Table S1 to S7

Figure S1

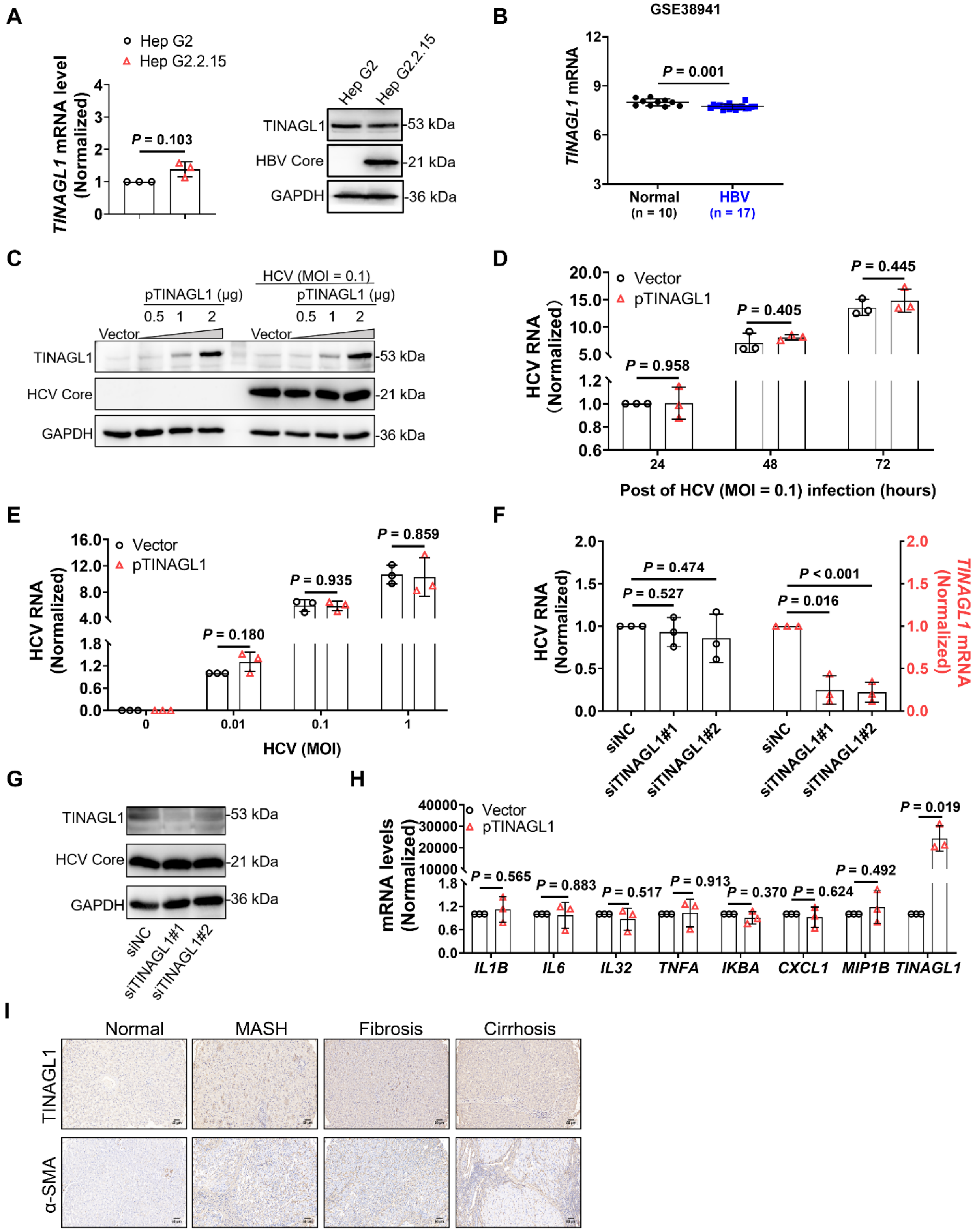
A
Hep G2
Hep G2.2.15
TINAGL1 mRNA level (Normalized)
P = 0.103
TINAGL1
HBV Core
GAPDH
53 kDa
21 kDa
36 kDa
B
GSE38941
TINAGL1 mRNA
P = 0.001
Normal (n = 10)
HBV (n = 17)
C
HCV (MOI = 0.1)
pTINAGL1 (μg)
0.5 1 2
Vector
HCV Core
D
Vector
pTINAGL1
HCV RNA (Normalized)
P = 0.958
P = 0.405
P = 0.445
24
48
72
Post of HCV (MOI = 0.1) infection (hours)
E
P = 0.180
P = 0.935
P = 0.859
HCV (MOI)
F
P = 0.474
P = 0.527
P < 0.001
P = 0.016
TINAGL1 mRNA (Normalized)
siNC
siTINAGL1#1
siTINAGL1#2
G
H
mRNA levels (Normalized)
P = 0.565
P = 0.883
P = 0.517
P = 0.913
P = 0.370
P = 0.624
P = 0.492
P = 0.019
IL1B
IL6
IL32
TNFA
IKBA
CXCL1
MIP1B
TINAGL1
I
Normal
MASH
Fibrosis
Cirrhosis
α-SMA

Figure S1. TINAGL1 has no effect on HCV replication, related to Figure 1. (A) mRNA and protein levels in Hep G2 and Hep G2.2.15 cells ($n = 3$). **(B)** *TINAGL1* mRNA in liver biopsies from Gene Expression Omnibus database (GSE38941). **(C)** Protein levels in Huh7.5 cells infected with HCV (MOI = 0.1) after being transfected with the TINAGL1 plasmid ($n = 3$). **(D-E)** RNA levels in Huh7.5 cells infected with HCV (MOI = 0.1) for 24, 48, and 72 hours **(D)** or infected with HCV (MOI = 0.01, 0.1, and 1) for 72 hours **(E)** after being transfected with the TINAGL1 plasmid ($n = 3$). **(F-G)** RNA **(F)** and protein **(G)** levels in Huh7.5 cells infected with HCV (MOI = 0.1) after being transfected with siRNA for TINAGL1 for 72 hours ($n = 3$). **(H)** mRNA levels in Huh7.5 transfected with the TINAGL1 plasmid ($n = 3$). (**I**) TINAGL1 expression shown with IHC staining (Scale bar: 50 μm) in a human liver tissue array. Data were expressed as mean ± standard deviation. *P* values were calculated by an unpaired two-tailed Student's t-test **(A-B, D-F, and H)**. *CXCL1*, C-X-C motif ligand 1; *IL1B*, interleukin-1B; *IL6*, interleukin 6; *IL32*, interleukin 32; *IKBA*, NF-Kappa-B inhibitor alpha; MASH, metabolic dysfunction-associated steatohepatitis; *MIP1B*, macrophage inflammatory protein-1 beta; NC, negative control; *TNFA*, tumor necrosis factor alpha; IHC, immunohistochemistry.

Figure S2

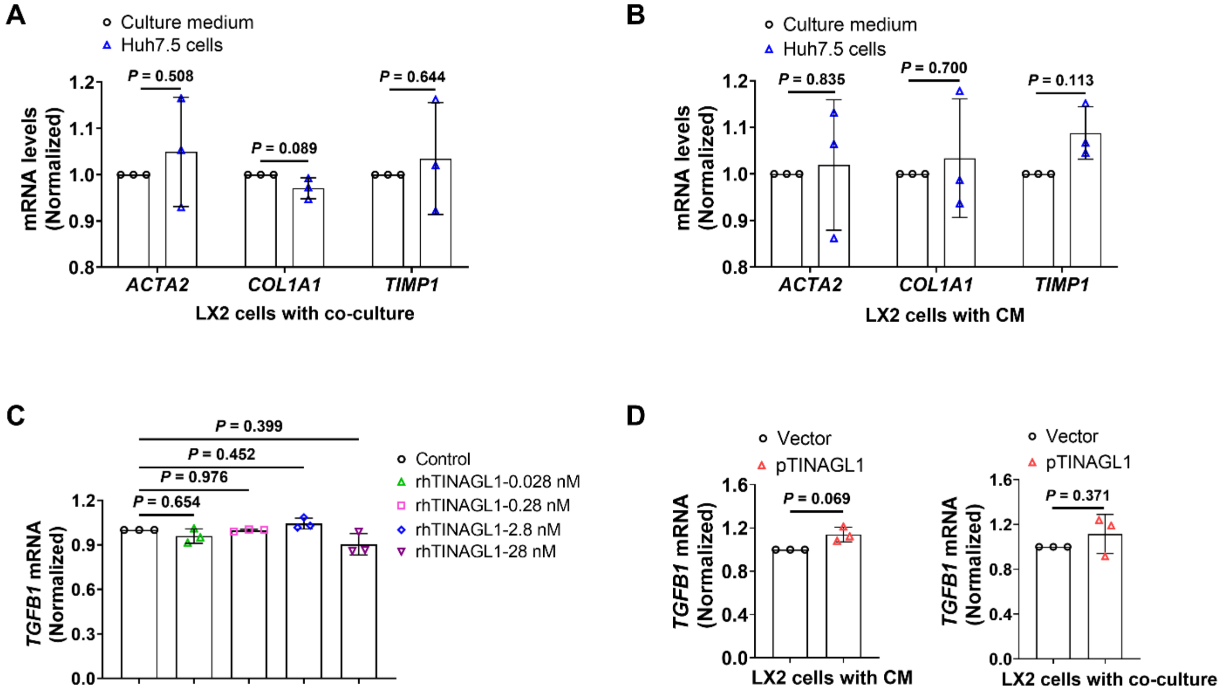

Figure S2. TINAGL1 has no effect on TGF-β1 expression, related to Figure 3. (A-B) mRNA levels in LX-2 cells co-cultured with native Huh7.5 cells **(A)** or their CM **(B)** (n = 3). **(C)** *TGFB1* mRNA in LX-2 cells treated with rhTINAGL1 for 48 h (n = 3). **(D)** *TGFB1* mRNA in LX-2 cells co-cultured with Huh7.5 cells transfected with the TINAGL1 plasmid or their CM (n = 3). Data were expressed as mean ± standard deviation. *P* values were calculated by an unpaired two-tailed Student's t-test **(A, B, D)** or one-way ANOVA **(C)** using Tukey's multiple comparisons test. *ACTA2* (α-SMA), alpha-smooth muscle actin; CM, conditioned medium; *COL1A1*, collagen type I alpha 1; rhTINAGL1, recombinant human tubulointerstitial nephritis antigen-like 1; *TIMP1*, tissue inhibitor of matrix metalloproteinase 1; *TGFB1*, transforming growth factor-beta1.

Figure S3

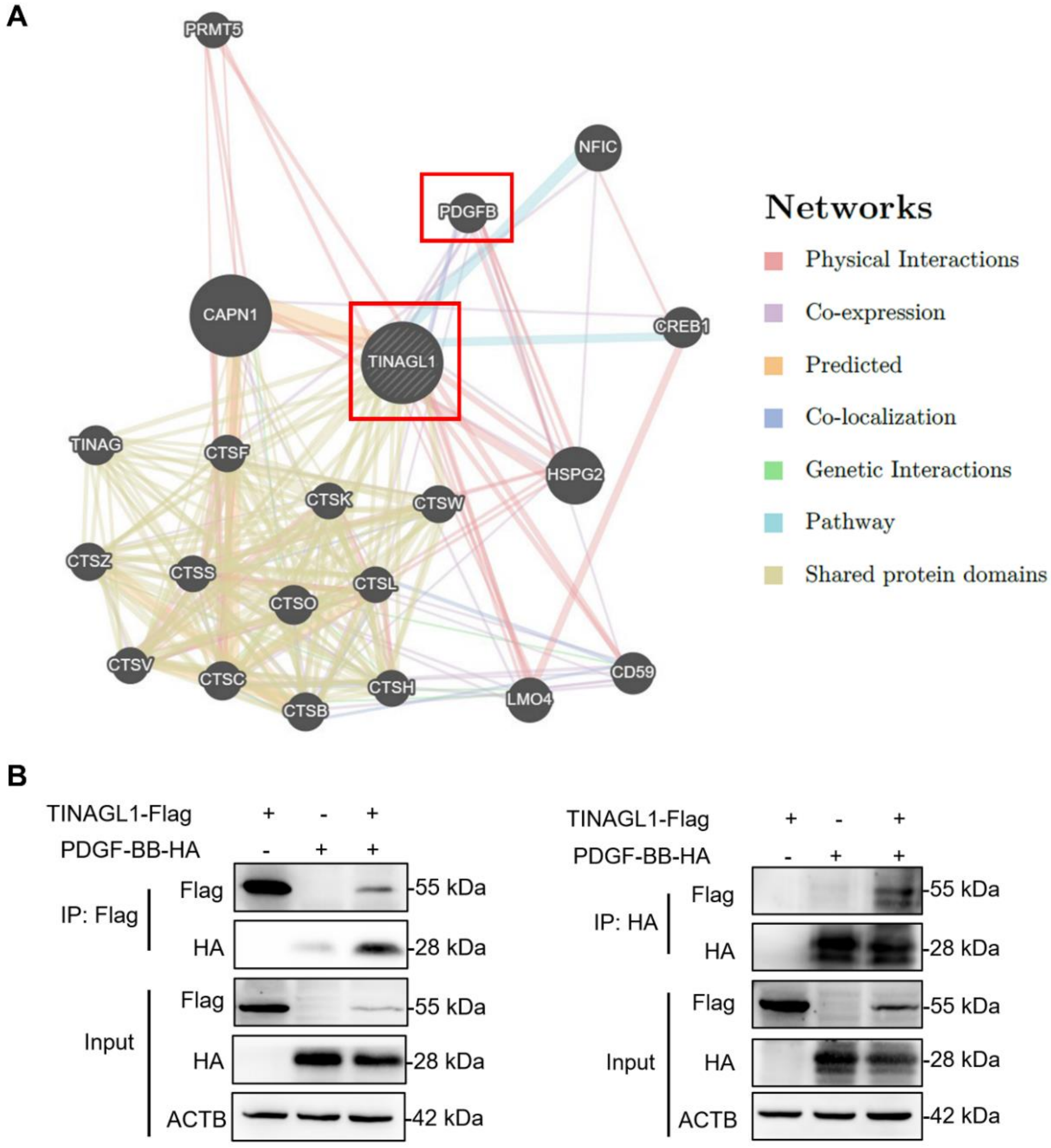

Figure S3. TINAGL1 activates HSCs by stabilizing PDGF-BB, related to Figure 4. (A) Interaction network of TINAGL1-regulated genes in GeneMANIA database, red frame shown the target proteins. **(B)** Interaction of TINAGL1 and PDGF-BB in Huh7.5 cells detected by co-immunoprecipitation. PDGF-BB, platelet-derived growth factor-BB; TINAGL1, tubulointerstitial nephritis antigen-like 1.

Figure S4

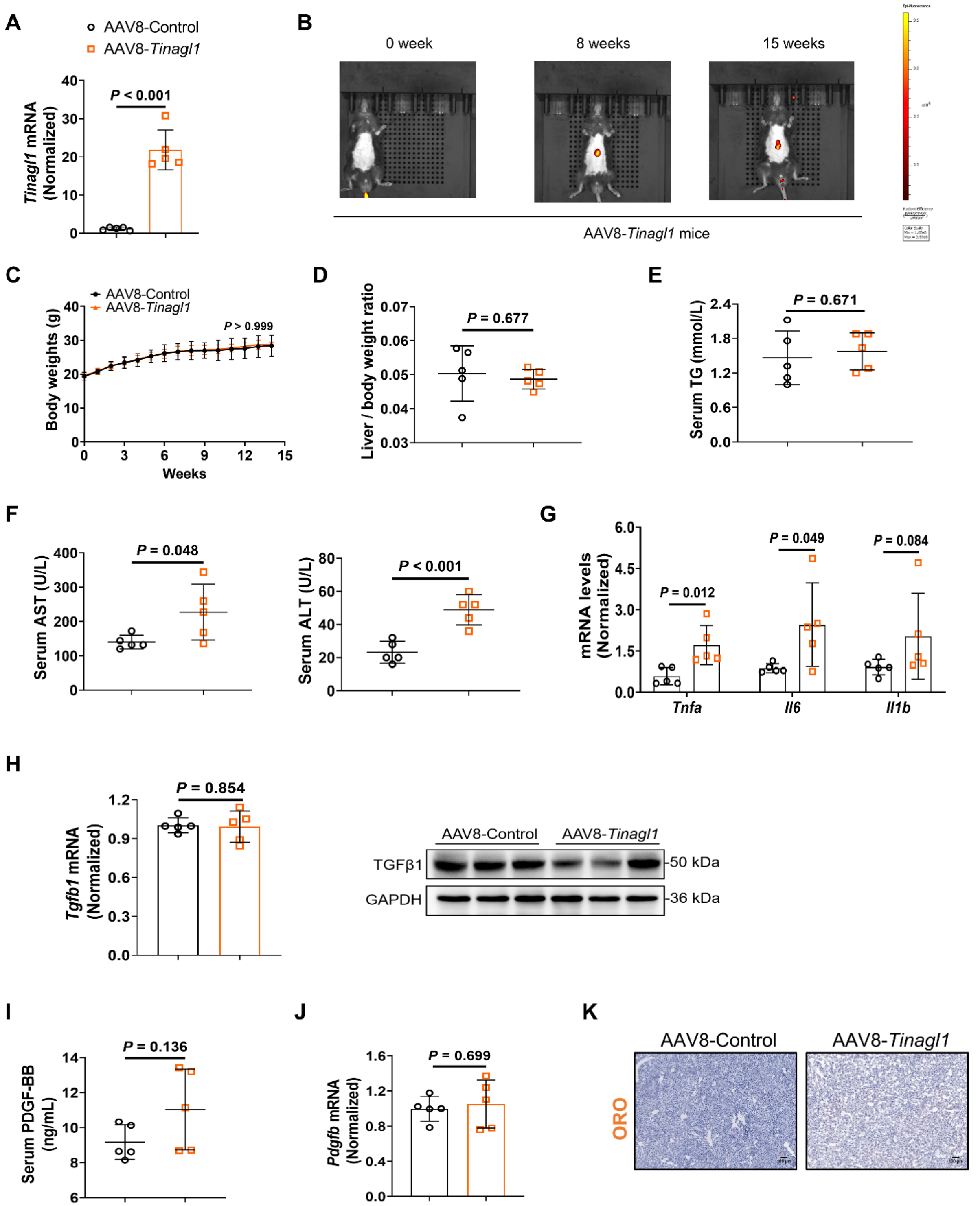

Figure S4. Liver-specific overexpression of TINAGL1 initiates liver fibrosis in mice, related

to Figure 5. (A) *Tinagl1* mRNA level in mouse livers (*n* = 5). **(B)** *In vivo* imaging of fluorescently labeled TINAGL1-His. **(C)** Body weight (*n* = 5). **(D)** Liver / body weight ratio (*n* = 5). **(E)** Serum TG (*n* = 5). **(F)** Serum AST and ALT (*n* = 5). **(G)** mRNA levels in mouse livers (*n* = 5). **(H)** mRNA and protein level of TGF-β1 in mouse livers (*n* = 5). **(I)** Serum PDGF-BB level quantified by ELISA at week fifteen (*n* = 5). **(J)** mRNA level of *Pdgfb* in mouse livers (*n* =5). **(K)** Oil Red O (ORO) staining. Data were expressed as mean ± standard deviation. *P* values were calculated by an unpaired two-tailed Student's t-test **(A, D-J)** or two-way ANOVA **(C)** using Bonferroni's multiple comparisons test. AST, aspartate aminotransferase; ALT alanine aminotransferase; PDGF-BB, platelet-derived growth factor-BB; TG, triglyceride; *Tgfb1*, transforming growth factor-beta1; *Il1b*, interleukin-1 beta; *Il6,* interleukin 6; *Tnfa*, tumor necrosis factor alpha.

Figure S5

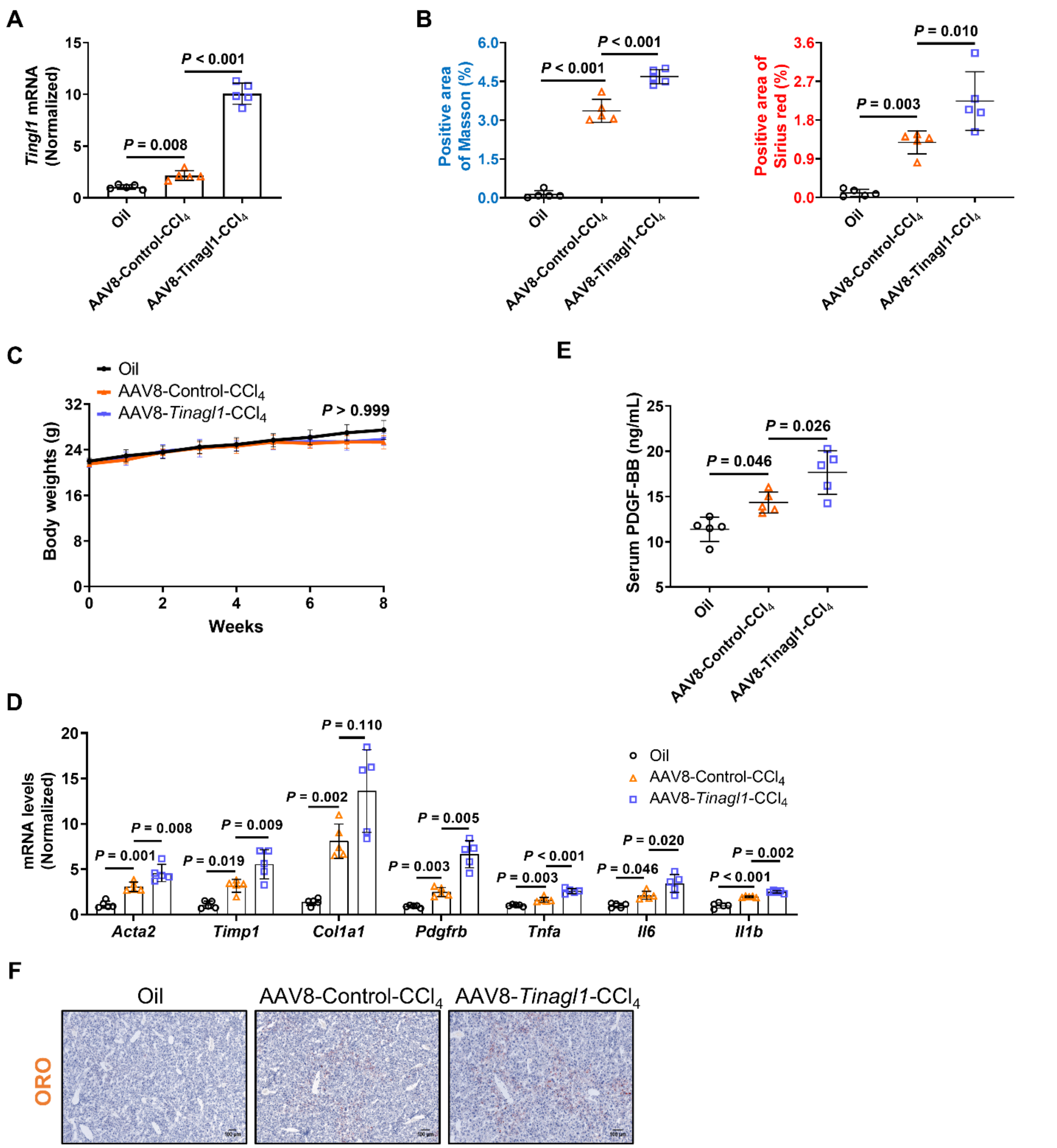

Figure S5. Liver-specific overexpression of TINAGL1 exacerbates liver fibrosis in mice induced by CCl_4, related to Figure 5. (A) *Tinagl1* mRNA level in mouse livers at week six (n = 5). **(B)** Quantification of Masson and Sirius red staining with Image J (n = 5). **(C)** Body weight (n

= 5). **(D)** mRNA levels in mouse livers (*n* = 5). **(E)** Serum PDGF-BB levels in mice quantified by ELISA (*n* = 5). **(F)** Oil Red O (ORO) staining. Data were expressed as mean ± standard deviation. *P* values were calculated by one-way ANOVA **(A-B, D-E)** using Tukey's multiple comparisons test or two-way ANOVA **(C)** using Bonferroni's multiple comparisons test. *Acta2* (α-SMA), alpha-smooth muscle actin; *Col1a1*, collagen type I alpha 1; PDGF-BB, platelet-derived growth factor-BB; *Pdgfrb*, platelet-derived growth factor receptor beta; *Timp1*, tissue inhibitor of matrix metalloproteinase 1; *Il1b*, interleukin-1 beta; *Il6,* interleukin 6; *Tnfa*, tumor necrosis factor alpha.

Figure S6

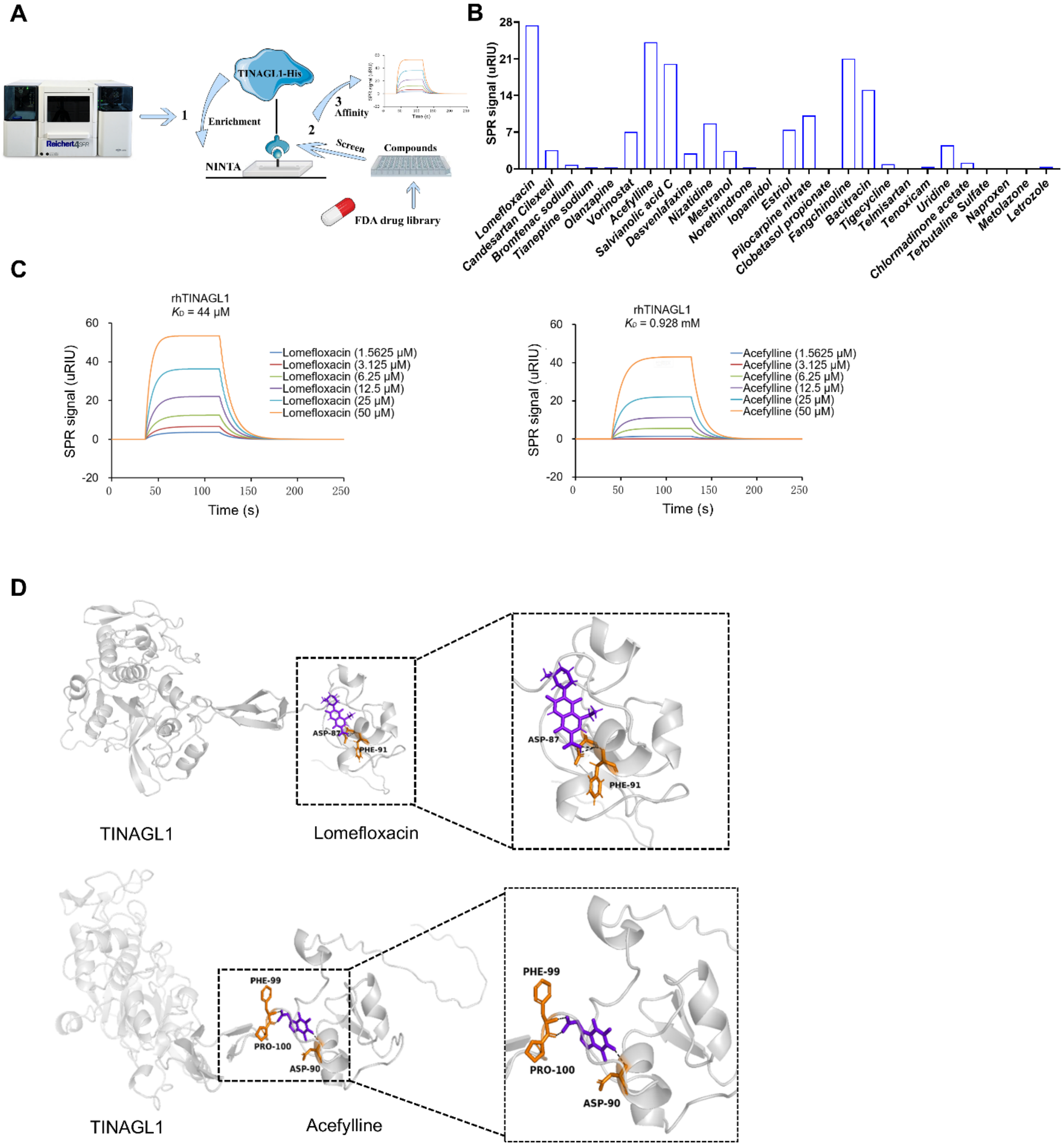

Figure S6. Compounds bind directly to TINAGL1 protein. (A) Drug screening schematic of compounds that bind to TINAGL1 protein using SPR technology. **(B)** SPR signal values for binding of some drugs (10 μM) to TINAGL1 protein. **(C)** Kinetic profiles of Lomefloxacin (left) or

Acefylline (right) binding to TINAGL1 protein. **(D)** Possible binding mode of Lomefloxacin and Acefylline with TINAGL1. SPR, surface plasmon resonance.

Figure S7

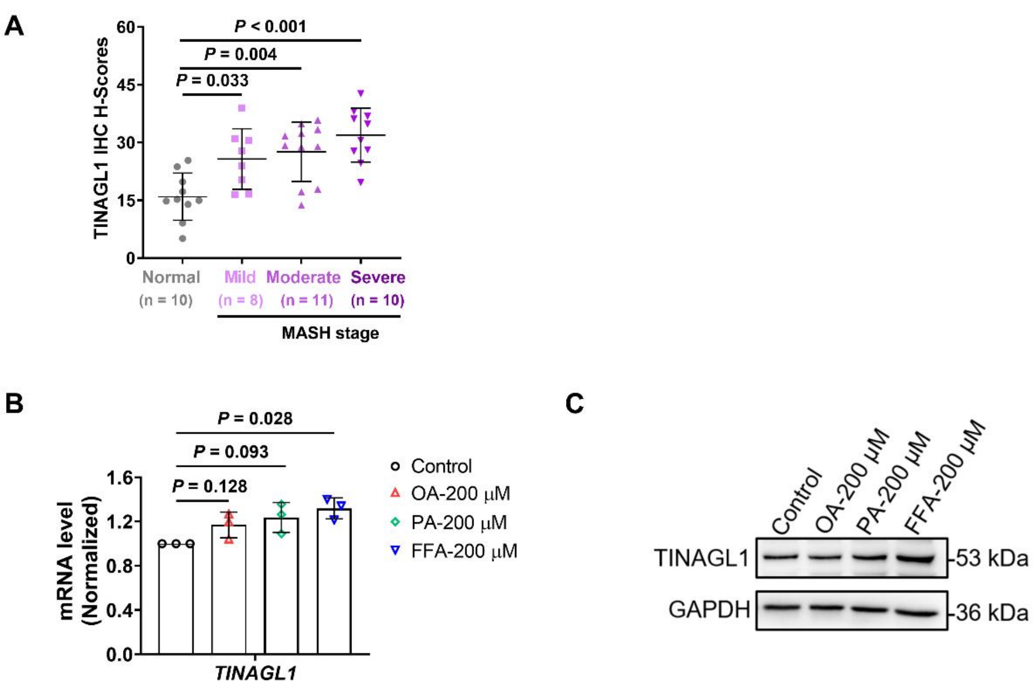

Figure S7. The expression of TINAGL1 in the progression of MASH. (A) The protein level of TINAGL1 in a human liver tissue array from patients with MASH. **(B-C)** mRNA **(B)** and protein **(C)** levels in Hep G2 cells treated with 200 μM of OA, PA, and FFA (n = 3). Data were expressed as mean ± standard deviation. P values were calculated by one-way ANOVA (**A, B**) using Tukey's multiple comparisons test. FFA, free fatty acid; OA, oleic acid; PA, palmitic acid.

Table S1. Patient information in D107Lv01 and H-Score immunohistochemical analysis

Location	Organ	Age	Gender	Pathologic diagnosis	Ishak score	H-Score
A1	liver	68	female	liver fibrosis (stage I)	2	13.2222
A2	liver	55	female	liver fibrosis (stage I)	1	26.8674
A3	liver	57	female	liver fibrosis (stage I)	2	37.6863
A4	liver	53	female	liver fibrosis (stage I)	1	36.7499
A5	liver	39	female	liver fibrosis (stage I)	2	39.9817
A6	liver	52	male	liver fibrosis (stage I)	2	20.9729
A7	liver	50	male	liver fibrosis (stage I)	2	24.4086
A8	liver	43	male	liver fibrosis (stage I)	2	32.9988
A9	liver	38	male	liver fibrosis (stage I)	2	34.5407
A10	liver	68	male	liver fibrosis (stage II)	3	41.3920
A11	liver	63	female	liver fibrosis (stage II)	2	32.6358
A12	liver	57	female	liver fibrosis (stage II)	3	44.3378
A13	liver	56	male	liver fibrosis (stage II)	2	43.1202
A14	liver	59	male	liver fibrosis (stage II)	2	41.2530
A15	liver	48	male	liver fibrosis (stage II)	2	39.5402
A16	liver	51	male	liver fibrosis (stage II)	3	32.4689
A17	liver	59	male	liver fibrosis (stage II)	3	17.2719
B1	liver	63	male	liver fibrosis (stage II)	3	24.8646
B2	liver	62	male	liver fibrosis (stage II)	3	30.6060
B3	liver	43	female	liver fibrosis (stage II)	3	28.8062
B4	liver	74	female	liver fibrosis (stage II)	3	35.0800

B5	liver	57	female	liver fibrosis (stage II)	3	36.2163
B6	liver	63	male	liver fibrosis (stage II)	3	33.0657
B7	liver	72	male	liver fibrosis (stage II)	3	30.6227
B8	liver	46	male	liver fibrosis (stage II)	3	23.5047
B9	liver	50	male	liver fibrosis (stage II)	3	34.5238
B10	liver	44	male	liver fibrosis (stage II)	3	39.4257
B11	liver	56	male	liver fibrosis (stage II)	4	42.1239
B12	liver	57	male	liver fibrosis (stage II)	4	46.6140
B13	liver	50	male	liver fibrosis (stage II)	3	39.6901
B14	liver	46	male	liver fibrosis (stage II)	4	38.5128
B15	liver	59	male	liver fibrosis (stage II)	3	37.0234
B16	liver	46	male	liver fibrosis (stage II)	4	36.7568
B17	liver	36	male	liver fibrosis (stage II)	4	27.9281
C1	liver	59	male	liver fibrosis (stage II)	3	23.2973
C2	liver	45	female	liver fibrosis (stage II)	3	23.6421
C3	liver	40	male	liver fibrosis (stage II)	4	38.4146
C4	liver	42	male	liver fibrosis (stage II)	3	42.1976
C5	liver	67	male	liver fibrosis (stage III)	4	32.6870
C6	liver	47	female	liver fibrosis (stage III)	4	30.9091
C7	liver	63	male	liver fibrosis (stage III)	3	23.9447
C8	liver	52	male	liver fibrosis (stage III)	4	3.2338
C9	liver	53	male	liver fibrosis (stage III)	3	33.6416
C10	liver	28	male	liver fibrosis (stage III)	3	35.4539

C11	liver	32	male	liver fibrosis (stage III)	4	38.2304
C12	liver	55	male	liver fibrosis (stage III)	4	32.7864
C13	liver	60	male	liver fibrosis (stage III)	4	37.7111
C14	liver	68	female	liver fibrosis (stage III)	4	43.2944
C15	liver	47	male	liver fibrosis (stage III)	4	35.6448
C16	liver	46	female	liver fibrosis (stage III)	4	3.2758
C17	liver	58	male	liver fibrosis (stage III)	4	33.6328
D1	liver	39	male	liver fibrosis (stage III)	4	27.1145
D2	liver	46	male	liver fibrosis (stage III)	4	31.1129
D3	liver	54	male	liver fibrosis (stage III)	4	26.4066
D4	liver	60	male	liver fibrosis (stage III)	4	39.3647
D5	liver	68	female	liver fibrosis (stage III)	4	28.6999
D6	liver	70	male	liver fibrosis (stage III)	4	36.0949
D7	liver	65	male	liver fibrosis (stage III)	4	28.2150
D8	liver	52	male	liver fibrosis (stage III)	4	25.2413
D9	liver	51	male	liver fibrosis (stage III)	4	24.3375
D10	liver	64	male	liver fibrosis (stage III)	4	21.9227
D11	liver	32	male	liver fibrosis (stage III)	4	30.9480
D12	liver	33	male	liver fibrosis (stage III)	4	32.7767
D13	liver	47	female	liver fibrosis (stage III)	4	25.2703
D14	liver	54	male	liver fibrosis (stage III)	4	37.4842
D15	liver	60	female	liver fibrosis (stage III)	4	39.4537
D16	liver	51	male	liver fibrosis (stage III)	4	38.1299

D17	liver	55	male	liver fibrosis (stage III)	4	30.7498
E1	liver	49	male	liver fibrosis (stage III)	4	22.5690
E2	liver	71	male	liver fibrosis (stage III)	4	34.0613
E3	liver	62	male	liver fibrosis (stage III)	4	29.6175
E4	liver	49	female	liver fibrosis (stage III)	4	32.3359
E5	liver	46	female	liver fibrosis (stage III)	4	32.2681
E6	liver	43	male	liver fibrosis (stage III)	4	33.0724
E7	liver	61	male	liver fibrosis (stage III)	5	25.7773
E8	liver	59	female	liver fibrosis (stage III)	5	25.4772
E9	liver	59	male	liver fibrosis (stage III)	5	29.0891
E10	liver	40	male	liver fibrosis (stage III)	5	29.7769
E11	liver	75	male	liver fibrosis (stage III)	5	28.4142
E12	liver	55	male	liver fibrosis (stage III)	5	23.5879
E13	liver	40	male	liver fibrosis (stage III)	5	31.6306
E14	liver	54	male	liver fibrosis (stage III)	5	22.0932
E15	liver	47	male	liver fibrosis (stage III)	5	20.1054
E16	liver	36	male	liver fibrosis (stage III)	5	30.1437
E17	liver	45	male	liver fibrosis (stage III)	5	19.7619
F1	liver	19	male	liver fibrosis (stage III)	5	24.7772
F2	liver	38	male	liver fibrosis (stage III)	4	4.8064
F3	liver	49	male	liver fibrosis (stage III)	4	33.4640
F4	liver	70	male	liver fibrosis (stage III)	4	36.9083
F5	liver	66	male	liver fibrosis (stage IV)	5	33.3337

F6	liver	56	male	liver fibrosis (stage IV)	5	26.9257
F7	liver	54	male	liver fibrosis (stage IV)	5	26.6547
F8	liver	49	female	liver fibrosis (stage IV)	5	27.8166
F9	liver	52	male	liver fibrosis (stage IV)	5	24.0380
F10	liver	56	male	liver fibrosis (stage IV)	5	30.7556
F11	liver	57	male	liver fibrosis (stage IV)	5	35.4586
F12	liver	45	male	liver fibrosis (stage IV)	5	28.4820
F13	liver	38	male	liver fibrosis (stage IV)	5	34.9991
F14	liver	64	male	liver fibrosis (stage IV)	6	28.2873
F15	liver	54	male	liver fibrosis (stage IV)	6	19.9402
F16	liver	43	female	inflammation (mild)	-	16.4731
F17	liver	74	male	inflammation (mild)	-	16.6232
G1	liver	52	male	inflammation (mild)	-	20.2704
G2	liver	63	male	inflammation (mild)	-	23.8998
G3	liver	57	female	inflammation (mild)	-	27.8027
G4	liver	64	male	inflammation (mild)	-	38.9708
G5	liver	33	male	inflammation (mild)	-	30.5774
G6	liver	64	male	inflammation (mild)	-	30.9619
G7	liver	76	male	inflammation (mild)	-	2.0227
G8	liver	43	male	inflammation (moderate)	-	34.8393
G9	liver	71	male	inflammation (moderate)	-	28.9349
G10	liver	42	male	inflammation (moderate)	-	32.3020
G11	liver	68	male	inflammation (moderate)	-	29.1624

G12	liver	34	female	inflammation (moderate)	-	33.3337
G13	liver	56	male	inflammation (moderate)	-	28.8146
G14	liver	45	male	inflammation (moderate)	-	35.7824
G15	liver	68	male	inflammation (moderate)	-	31.6546
G16	liver	63	male	inflammation (moderate)	-	17.8675
G17	liver	37	female	inflammation (moderate)	-	17.1633
H1	liver	65	male	inflammation (moderate)	-	13.6938
H2	liver	49	female	inflammation (moderate)	-	19.5932
H3	liver	43	male	inflammation (moderate)	-	24.6712
H4	liver	58	male	inflammation (moderate)	-	27.7078
H5	liver	57	male	inflammation (moderate)	-	30.5710
H6	liver	69	female	inflammation (moderate)	-	36.8005
H7	liver	57	male	inflammation (moderate)	-	38.1060
H8	liver	50	male	inflammation (moderate)	-	42.6723
H9	liver	57	male	inflammation (moderate)	-	28.0906
H10	liver	48	female	inflammation (moderate)	-	36.0959
H11	liver	57	female	inflammation (moderate)	-	34.7690
H12	liver	66	male	cirrhosis	-	44.7993
H13	liver	46	male	cirrhosis	-	40.1116
H14	liver	50	female	cirrhosis	-	35.0177
H15	liver	62	male	cirrhosis	-	25.7583
H16	liver	52	male	cirrhosis with steatosis	-	16.1989
H17	liver	47	male	cirrhosis	-	15.9356

I1	liver	53	male	cirrhosis	-	8.9353
I2	liver	57	female	cirrhosis with steatosis	-	13.4913
I3	liver	69	female	cirrhosis with steatosis	-	25.4892
I4	liver	48	male	cirrhosis	-	31.6548
I5	liver	51	male	cirrhosis	-	28.6824
I6	liver	66	male	cirrhosis	-	39.0532
I7	liver	54	female	cirrhosis	-	42.3739
I8	liver	50	male	cirrhosis	-	32.3964
I9	liver	56	male	cirrhosis	-	38.5455
I10	liver	46	male	cirrhosis	-	31.7928
I11	liver	52	male	cirrhosis	-	38.3738
I12	liver	58	male	cirrhosis	-	38.0049
I13	liver	50	male	cirrhosis	-	33.2002
I14	liver	63	female	cirrhosis	-	28.5899
I15	liver	73	male	cirrhosis	-	19.7783
I16	liver	52	male	cirrhosis	-	11.8258
I17	liver	58	female	cirrhosis	-	10.9895
J1	liver	44	male	cirrhosis	-	4.2474
J2	liver	59	male	cirrhosis	-	9.4892
J3	liver	63	male	cirrhosis	-	13.9982
J4	liver	54	male	cirrhosis	-	20.3364
J5	liver	53	male	cirrhosis	-	21.5947
J6	liver	68	male	cirrhosis	-	29.7777

J7	liver	38	male	cirrhosis	-	25.2262
J8	liver	16	male	normal	-	25.3508
J9	liver	45	male	normal	-	23.6936
J10	liver	23	male	normal	-	19.7892
J11	liver	23	male	normal	-	17.2289
J12	liver	40	male	normal	-	14.9651
J13	liver	56	female	normal	-	14.7993
J14	liver	35	male	normal	-	15.2506
J15	liver	21	female	normal	-	13.9430
J16	liver	40	female	normal	-	9.0924
J17	liver	17	male	normal	-	5.0511

A16, B8, B14, C1, C2, C8, C16, F2, and G7 were excluded due to sample damage during processing.

Table S2. Cytokines secreted by Huh7.5 cells transfected with TINAGL1 plasmid

Cytokines	Description	Fold change	Regulated
IL-12 p70	Interleukin-12 p70	1.246	Up
MCP-3 (CCL7)	C-C motif chemokine 7	1.246	Up
MDC (CCL22)	C-C motif chemokine 22	1.404	Up
FGF-9	Fibroblast growth factor 9	1.910	Up
IL-3	Interleukin-3	1.919	Up
IGFBP-4	Insulin-like growth factor-binding protein 4	1.966	Up
EGF	Epidermal growth factor	2.033	Up
MCP-4 (CCL13)	C-C motif chemokine 13	2.064	Up
CCL23	C-C motif chemokine 23	2.103	Up
IL-4	Interleukin-4	4.117	Up
LIGHT (TNFSF14)	Tumor necrosis factor ligand superfamily member 14	26.227	Up
GDNF	Glial cell line-derived neurotrophic factor	26.347	Up
LIF	Leukemia inhibitory factor	50.247	Up
BDNF	Brain-derived neurotrophic factor	66.461	Up
PDGF-BB	Platelet-derived growth factor BB	106.214	Up
I-309 (CCL1)	C-C motif chemokine 1	0.004	Down
Eotaxin (CCL11)	C-C motif chemokine 11	0.011	Down
IL-1β	Interleukin-1 beta	0.048	Down

Eotaxin-3 (CCL26)	C-C motif chemokine 26	0.191	Down
Flt-3 Ligand	Receptor-type tyrosine-protein kinase FLT3	0.470	Down
MIP1δ	C-C motif chemokine 15	0.480	Down
PARC (CCL18)	C-C motif chemokine 18	0.485	Down
IL-1α	Interleukin-1 alpha	0.612	Down
TGF-β1	Transforming growth factor beta-1	0.631	Down
ENA-78 (CXCL5)	C-X-C motif chemokine 5	0.636	Down
GCSF	Granulocyte colony-stimulating factor	0.642	Down
IL-2	Interleukin-2	0.649	Down
IP-10 (CXCL10)	C-X-C motif chemokine 10	0.657	Down
GM-CSF	Granulocyte-macrophage colony-stimulating factor	0.680	Down
Angiogenin	Angiogenin	0.684	Down
NAP-2 (CXCL7)	C-X-C motif chemokine 7	0.686	Down
IL-15	Interleukin-15	0.689	Down
TIMP1	Metalloproteinase inhibitor 1	0.708	Down
IGFBP1	Insulin-like growth factor-binding protein 1	0.712	Down
TNFβ	Tumor necrosis factor ligand superfamily member 1	0.716	Down
IL-6	Interleukin-6	0.722	Down
Osteopontin (SPP1)	Secreted phosphoprotein 1	0.735	Down

MIG (CXCL9)	C-X-C motif chemokine 9	0.742	Down
IGFBP2	Insulin-like growth factor-binding protein 2	0.744	Down
GRO (CXCL1)	C-X-C motif chemokine 1	0.746	Down
MIP1β (CCL4)	C-C motif chemokine 4	0.751	Down
MCP-1 (CCL2)	C-C motif chemokine 2	0.755	Down
IL-5	Interleukin-5	0.763	Down
GRO-α (CXCL1)	C-X-C motif chemokine 1	0.765	Down
MIF (CXCL9)	C-X-C motif chemokine 9	0.771	Down
TNF-α	Tumor necrosis factor α	0.772	Down
Osteoprotegerin	Tumor necrosis factor receptor superfamily member 11B	0.774	Down
TIMP-2	Metalloproteinase inhibitor 2	0.775	Down
IL-7	Interleukin-7	0.779	Down
IL-13	Interleukin-13	0.797	Down
IFN-γ	Interferon gamma	0.802	Down
VEGF	Vascular endothelial growth factor	0.804	Down
IL-8	Interleukin-8	0.816	Down
IL-10	Interleukin-10	0.824	Down
NT-3	Neurotrophin-3	0.856	No difference
TGF-β2	Transforming growth factor beta-2	0.884	No difference
MIP-3α (CCL20)	C-C motif chemokine 20	0.905	No difference
GCP-2 (CXCL6)	C-X-C motif chemokine 6	0.918	No difference

FGF-6	Fibroblast growth factor 6	0.980	No difference
MCP-2 (CCL8)	C-C motif chemokine 8	1.006	No difference
RANTES (CCL5)	C-C motif chemokine 5	1.006	No difference
SCF	Stem cell factor	1.006	No difference
SDF-1 (CXCL12)	C-X-C motif chemokine 12	1.006	No difference
TARC (CCL17)	C-C motif chemokine 17	1.006	No difference
IGF-I	Insulin-like growth factor I	1.006	No difference
Oncostatin M	Oncostatin-M	1.006	No difference
Thrombopoietin	Thrombopoietin	1.006	No difference
BLC (CXCL13)	C-X-C motif chemokine 13	1.006	No difference
Eotaxin-2 (CCL24)	C-C motif chemokine 24	1.006	No difference
FGF-4	Fibroblast growth factor 4	1.006	No difference
Fractalkine (CX3CL1)	C-X3-C motif chemokine 1	1.006	No difference
HGF	Hepatocyte growth factor	1.006	No difference
IGFBP-3	Insulin-like growth factor-binding protein 3	1.006	No difference
IL-16	Interleukin-16	1.006	No difference
NT-4	Neurotrophin-4	1.006	No difference
TGF-β3	Transforming growth factor beta-3	1.006	No difference
PIGF	Placenta growth factor	1.010	No difference
FGF-7	Fibroblast growth factor 7	1.103	No difference
MCSF	Macrophage colony-stimulating factor 1	1.141	No difference

Table S3. Primers for qRT-PCR

GENE	Primer（5'-3'）	
Human *GAPDH*	Forward	CGGAGTCAACGGATTTGGTCGTAT
	Reverse	AGCCTTCTCCATGGTGGTGAAGAC
Human *MTMR11*	Forward	GCTGCTCAGAGTTGGTTTTGA
	Reverse	CCCCGAATACTGTTGGGCTT
Human *MKNK2*	Forward	CAGGGACACAGGAACGTCCTA
	Reverse	GGGCGATGCCTTTGTTATGC
Human *TINAGL1*	Forward	TCTTCCTCGGTCATGAACATGCA
	Reverse	TTGCCTTGGTCAAGAGGCTCATG
Human *CYP2S1*	Forward	GATGGACGGTTCAGGAAGCATG
	Reverse	GGAGAAGGCTTGTAGGATGGTG
Human *OAS1*	Forward	AGTTGACTGGCGGCTATAAAC
	Reverse	GTGCTTGACTAGGCGGATGAG
Human *ST6GALNAC1*	Forward	CACAGCCAAGACGCTCATTC
	Reverse	CCTTTCTGTCTCGTCCTTGTTG
Human *NOX1*	Forward	GCACACCTGTTTAACTTTGACTG
	Reverse	GGACTGGATGGGATTTAGCCA
Human *CDS1*	Forward	AAGGCATGATATGGTTCCTTGTT
	Reverse	TCACTTCGGTATTCCACTGGG
Human *TFEC*	Forward	TTGGATGTGTATAGCGGTGAAC
	Reverse	GGTAGACTACTTGGACAAGAAGC

Human *CHI3L1*	Forward	GAAGACTCTCTTGTCTGTCGGA
	Reverse	AATGGCGGTACTGACTTGATG
Human *IL2RG*	Forward	GTGCAGCCACTATCTATTCTCTG
	Reverse	GTGAAGTGTTAGGTTCTCTGGAG
Human *MICAL1*	Forward	GGCACTCGGTGCTAAGAAGTT
	Reverse	CCCCAGTGAATTTCCACCCC
Human *ANXA1*	Forward	GCGGTGAGCCCCTATCCTA
	Reverse	TGATGGTTGCTTCATCCACAC
Human *ERCC2*	Forward	AGAAGGTGATTGAAGAGCTTCG
	Reverse	ACCTCAGGGTGAATACACAAGT
Human *PHGR1*	Forward	CAGGACACAAGATGGCAAGC
	Reverse	TGGCCTCTAGGAGGCTGTTTA
Human *SLPI*	Forward	AGCGTGACTTGAAGTGTTGCATG
	Reverse	GAAAGGACCTGGACCACACAGA
Human *PI3*	Forward	CACGGGAGTTCCTGTTAAAGG
	Reverse	TCTTTCAAGCAGCGGTTAGGG
Human *AREG*	Forward	GTGGTGCTGTCGCTCTTGATA
	Reverse	CCCCAGAAAATGGTTCACGCT
Human *KCNH4*	Forward	GTACTGTCCTACACCGACTGA
	Reverse	GCAACGTAGAAGGTGGCAAG
Human *ST8STA2*	Forward	CACAGCTTCGTCATCAGGTG
	Reverse	GTTCATGGTTACCAGGTCTGTC

Human *FBLIM1*	Forward	TCAATGGAGACATCTGTGCCT
	Reverse	CAGGTGCGGCACGTGAAGCACT
Human *IL6*	Forward	CAGGAGCCCAGCTATGAACT
	Reverse	AGCAGGCAACACCAGGAG
Human *IL1B*	Forward	GCACCTTCTTTCCCTTCATCTTTG
	Reverse	GCTTTTTTGCTGTGAGTCCCG
Human *IL32*	Forward	TGGCGGCTTATTATGAGGAGC
	Reverse	CTCGGCACCGTAATCCATCTC
Human *TNFA*	Forward	CAGCCTCTTCTCCTTCCTGAT
	Reverse	GCCAGAGGGCTGATTAGAGA
Human *IKBA*	Forward	CTCCGAGACTTTCGAGGAAATAC
	Reverse	GCCATTGTAGTTGGTAGCCTTCA
Human *CXCL1*	Forward	CTGGCGGATCCAAGCAAAT
	Reverse	CATTCCCCTGCCTTCACAAT
Human *MIP1B*	Forward	CAGCGCTCTCAGCACCAATGG
	Reverse	GATCAGCACAGACTTGCTTGCTTC
Human *ACTA2*	Forward	GAGTTACGAGTTGCCTGATGG
	Reverse	GATGCTGTTGTAGGTGGTTTCA
Human *COL1A1*	Forward	TCTGGCGCTCCCATGGCTCT
	Reverse	GCCCTGCGGCACAAGGGATT
Human *TIMP1*	Forward	TGTTGTTGCTGTGGCTGATAGC
	Reverse	TCTGGTGTCCCCACGAACTT

Human *PDGFRB*	Forward	AGACACGGGAGAATACTTTTGC
	Reverse	AGTTCCTCGGCATCATTAGGG
Human *PDGFB*	Forward	CTCGATCCGCTCCTTTGATGA
	Reverse	CGTTGGTGCGGTCTATGAG
Human *TGFB1*	Forward	CCGACTACTACGCCAAGGAG
	Reverse	TGAGGTATCGCCAGGAATTG
HCV	Forward	CGGGAGAGCCATAGTGGTCTGCG
	Reverse	CTCGCAAGCACCCTATCAGGCAGTA
	Probe	FAM-5′-AGGCCTTGTGGTACTGCCT-3′-TAMRA
GAPDH [a]	Forward	CGGAGTCAACGGATTTGGTCGTAT
	Reverse	AGCCTTCTCCATGGTGGTGAAGAC
	Probe	FAM-5′-CCGTCAAGGCTGAGAACGG-3′-TAMRA
Mouse *Gapdh*	Forward	CTCTGGAAAGCTGTGGCGTGATG
	Reverse	ATGCCAGTGAGCTTCCCGTTCAG
Mouse *Tinagl1*	Forward	CCCGACTTCTGGGACTTCTG
	Reverse	AGTAGGTTCCGAAGACTGGGT
Mouse *Acta2*	Forward	GAGCATCCGACACTGCTGAC
	Reverse	GCACAGCCTGAATAGCCACA
Mouse *Col1a1*	Forward	TAGGCCATTGTGTATGCAGC
	Reverse	ACATGTTCAGCTTTGTGGACC
Mouse *Timp1*	Forward	AGGTGGTCTCGTTGATTTCT
	Reverse	GTAAGGCCTGTAGCTGTGCC

Mouse *Pdgfrb*	Forward	TTGCCTTACGACTCCACCTG
	Reverse	TAGATGGGCCCTCCTTTGGT
Mouse *Pdgfb*	Forward	TTCCTCATGTGCCCTTCAGT
	Reverse	TGCTTGGCTGGAGTGGAATA
Mouse *Il6*	Forward	CCATCCAGTTGCCTTCTTGG
	Reverse	TGCAAGTGCATCATCGTTGT
Mouse *Tnfa*	Forward	CCAAAGGGATGAGAAGTTCC
	Reverse	CTCCACTTGGTGGTTTGCTA
Mouse *Il1b*	Forward	TGTCTGAAGCAGCTATGGCAAC
	Reverse	CTGCCTGAAGCTCTTGTTGATG

[a] internal reference for HCV quantification.

Table S4. siRNA sequences

Gene	siRNA sequence
TINAGL1#1	CCATCTTCCTCGGTCATGA
TINAGL1#2	CCGATCGTGTCTCAATCCA
PDGFRB	CAACGAGTCTCCAGTGCTA

Table S5. Primary antibody

Antibody	Manufacturers	Cat No.
GAPDH Rabbit Polyclonal antibody	Proteintech	10494-1-AP
TINAGL1 Polyclonal antibody	Proteintech	12077-1-AP
Hepatitis C Virus Core 1b Mouse monoclonal antibody	Abcam	ab2740
Alpha Smooth Muscle Actin Mouse Monoclonal antibody	Abcam	ab7817
TIMP1 Mouse Monoclonal antibody	Santa cruz	sc-365905
COL1A1 Rabbit Monoclonal antibody	Cell signaling technology	84336
PDGF Receptor β Rabbit Monoclonal antibody	Cell signaling technology	3169
PDGF-B Rabbit Monoclonal antibody	Abcam	ab178409
Actin Rabbit Monoclonal Antibody	Huabio	ET1702-52
DYKDDDDK Tag Mouse Monoclonal antibody	Proteintech	66008-4-Ig
HA-Tag Rabbit Monoclonal antibody	Cell signaling technology	3724

Table S6. Primers for plasmid construction

GENE		Primer（5'-3'）
Human *TINAGL1*	Forward	GGGGTACCCCATGTGGCGATGTCCACTGGG
	Reverse	GCTCTAGAGCTCAGTGATGACCCATGTCCTC
Human *TINAGL1-Flag*	Forward	GGGGTACCCCATGTGGCGATGTCCACTGGG
	Reverse	GCTCTAGAGCTCACTTGTCATCGTCGTCCTTGTA GTCGTGATGACCCATGTCCTCCA

Table S7. Twenty-one consensus genes in HCV-infected Huh7.5 cells for one to three months (Fold change ≥ 2)

Genes	Description	Fold change compared to the control			Regulated
		1 month	2 months	3 months	
MTMR11	Myotubularin related protein 11	2.5	4.0	3.6	Up
MKNK2	MAP kinase-interacting serine/threonine-protein kinase 2	2.7	3.9	2.5	Up
TINAGL1	Tubulointerstitial nephritis antigen-like 1	2.2	2.2	2.7	Up
CYP2S1	Cytochrome P450 2S1	2.2	11	5	Up
OAS1	2'-5'-oligoadenylate synthase 1	2.4	8.5	8.1	Up
TFEC	Transcription factor EC	2.4	4.5	4.2	Up
NOX1	NADPH oxidase 1	2.6	6.1	2.8	Up
CDS1	Phosphatidate cytidylyltransferase 1	2.2	5.7	2.7	Up
IL2RG	Cytokine receptor common subunit gamma	2.0	4.1	2.6	UP
MICAL1	[F-actin]-monooxygenase MICAL1	2.0	5.4	2.8	UP
CHI3L1	Chitinase-3-like protein 1	2.7	2.2	2.1	UP

ANXA1	Annexin A1	2.9	3.5	2.4	UP
PI3	Elafin	2.9	4.0	3.9	UP
ST8SIA2	Alpha-2,8-sialyltransferase 8B	2.2	3.4	3.0	Up
SLPI	Antileukoproteinase	2.9	4.7	5.6	UP
ERCC2	General transcription and DNA repair factor IIH helicase subunit XPD	2.1	2.4	2.0	Up
PHGR1	Proline, histidine and glycine-rich protein 1	2.7	6.3	9.8	Up
FBLIM1	Filamin-binding LIM protein 1	2.2	4.4	2.1	UP
AREG	Amphiregulin	2.1	4.4	3.2	UP
ST6GALNAC1	Alpha-N acetylgalactosaminide alpha-2,6-sialyltransferase 1	3.3	9.1	6.1	UP
KCNH4	Potassium voltage-gated channel subfamily H member 4	2.4	2.1	3.0	UP
